# COVID-19 Convalescent Plasma for the Treatment of Immunocompromised Patients: A Systematic Review and Meta-analysis

**DOI:** 10.1001/jamanetworkopen.2022.50647

**Published:** 2023-01-12

**Authors:** Jonathon W. Senefeld, Massimo Franchini, Carlo Mengoli, Mario Cruciani, Matteo Zani, Ellen K. Gorman, Daniele Focosi, Arturo Casadevall, Michael J. Joyner

**Affiliations:** 1 Department of Anesthesiology and Perioperative Medicine, Mayo Clinic, Rochester, Minnesota; 2 Division of Transfusion Medicine, Carlo Poma Hospital, Mantua, Italy; 3 North-Western Tuscany Blood Bank, Pisa University Hospital, Pisa, Italy; 4 Department of Molecular Microbiology and Immunology, Johns Hopkins Bloomberg School of Public Health, Baltimore, Maryland

## Abstract

**Importance:**

Patients who are immunocompromised have increased risk for morbidity and mortality associated with coronavirus disease 2019 (COVID-19) because they less frequently mount antibody responses to vaccines. Although neutralizing anti-spike monoclonal-antibody treatment has been widely used to treat COVID-19, evolutions of SARS-CoV-2 have been associated with monoclonal antibody-resistant SARS-CoV-2 variants and greater virulence and transmissibility of SARS-CoV-2. Thus, the therapeutic use of COVID-19 convalescent plasma has increased on the presumption that such plasma contains potentially therapeutic antibodies to SARS-CoV-2 that can be passively transferred to the plasma recipient.

**Objective:**

To assess the growing number of reports of clinical experiences of patients with COVID-19 who are immunocompromised and treated with specific neutralizing antibodies via COVID-19 convalescent plasma transfusion.

**Data Sources:**

On August 12, 2022, a systematic search was performed for clinical studies of COVID-19 convalescent plasma use in patients who are immunocompromised.

**Study Selection:**

Randomized clinical trials, matched cohort studies, and case report or series on COVID-19 convalescent plasma use in patients who are immunocompromised were included. The electronic search yielded 462 unique records, of which 199 were considered for full-text screening.

**Data Extraction and Synthesis:**

The study followed the Preferred Reporting Items for Systematic Reviews and Meta-Analyses guidelines. Data were extracted by 3 independent reviewers in duplicate and pooled.

**Main Outcomes and Meaures:**

The prespecified end point was all-cause mortality after COVID-19 convalescent plasma transfusion; exploratory subgroup analyses were performed based on putative factors associated with the potential mortality benefit of convalescent plasma.

**Results:**

This systematic review and meta-analysis included 3 randomized clinical trials enrolling 1487 participants and 5 controlled studies. Additionally, 125 case series or reports enrolling 265 participants and 13 uncontrolled large case series enrolling 358 participants were included. Separate meta-analyses, using models both stratified and pooled by study type (ie, randomized clinical trials and matched cohort studies), demonstrated that transfusion of COVID-19 convalescent plasma was associated with a decrease in mortality compared with the control cohort for the amalgam of both randomized clinical trials and matched cohort studies (risk ratio [RR], 0.63 [95% CI, 0.50-0.79]).

**Conclusions and Relevance:**

These findings suggest that transfusion of COVID-19 convalescent plasma is associated with mortality benefit for patients who are immunocompromised and have COVID-19.

## Key Points

**Question:**

What is the pooled evidence regarding the potential mortality benefit associated with transfusion of convalescent plasma in patients who are immunocompromised and have COVID-19?

**Findings:**

In this systematic review and meta-analysis including 3 randomized clinical trials, 5 matched cohort studies, 13 uncontrolled large case series, and 125 case report series, transfusion of convalescent plasma was associated with a mortality benefit in patients who are immunocompromised and have COVID-19.

**Meaning:**

These findings suggest that transfusion of COVID-19 convalescent plasma may be associated with a mortality benefit for patients who are immunocompromised who are susceptible to refractory infection.

## Teaser

This systematic review and meta-analysis assesses reports of clinical experiences of patients with COVID-19 and immunosuppression who were treated with specific neutralizing antibodies via COVID-19 convalescent plasma transfusion.

## Introduction

In December 2019, SARS-CoV-2 emerged in Wuhan, China,^1,2^ causing COVID-19. COVID-19 rapidly spread across the globe leading to a pandemic with nearly 642 million infected people worldwide and 6.6 million deaths as of December 2022.^3^ Many treatments, including antiviral, anticoagulant, and anti-inflammatory agents, have been tested in patients with COVID-19, often with controversial results.^4^ The passive transfer of anti–SARS-CoV-2 neutralizing antibodies from the plasma of recently recovered individuals (COVID-19 convalescent plasma) to patients with severe COVID-19 was among the first therapies used.^5,6,7^ There is now substantial evidence suggesting that such antibody-based therapy, when administered early in the disease course (ie, within 72 hours since the onset of symptoms) and with high titers of neutralizing antibodies, is associated with a clinical benefit— including decreases in incidences of disease progression, hospitalization, and mortality.^8,9^

Although neutralizing anti-spike monoclonal-antibody treatment has been widely used to manage COVID-19, evolutions of SARS-CoV-2 have been associated with monoclonal antibody-resistant SARS-CoV-2 variants,^10,11,12^ and greater virulence and transmissibility in emerging SARS-CoV-2 variants.^13,14,15^ By contrast, COVID-19 convalescent plasma appears to have maintained clinical efficacy over time with emerging SARS-CoV-2 variants due to heterogenous, broad spectrum of neutralizing antibodies and widespread availability.^16,17^ Thus, there has been a renewed interest in the clinical use of COVID-19 convalescent plasma, particularly for patients who are immunocompromised, who are not able to mount a sufficiently protective antibody response against the virus, and who have contraindications or adverse effects from small molecule antivirals.^18,19^ These patients who are immunocompromised are at higher risk for morbidity and mortality associated with COVID-19.^20^ A few controlled studies and a number of case reports and case series have shown a clinical benefit from COVID-19 convalescent plasma among patients with hematological or solid cancer or other underlying causes of immunosuppression. Thus, on January 2022, the US Food and Drug Administration (FDA) revised the Emergency Use Authorization (EUA) of COVID-19 convalescent plasma to include patients who are hospitalized with impaired humoral immunity.^21^ In this context, we performed a systematic review to summarize the growing number of reports of clinical experiences of patients with COVID-19 with immunosuppression who were treated with specific neutralizing antibodies via COVID-19 convalescent plasma transfusion.

## Methods

This systematic review and meta-analysis followed the recommendations in the *Cochrane Handbook for Systematic Review of Interventions* and reported findings according to the Preferred Reporting Items for Systematic Reviews and Meta-analyses (PRISMA) reporting guideline (eTable 1 in the Supplement). The study protocol has been registered in PROSPERO (CRD42022316321); all changes to the protocol are reported in the Methods section. In accordance with the Code of Federal Regulations, 45 CFR 46.102, this study was exempt from obtaining institutional review board approval from Mayo Clinic and the requirement to obtain informed patient consent because it is a secondary use of publicly available data sets.

### Information Sources

The purpose of this systematic review was to investigate the impact of COVID-19 convalescent plasma on COVID-19 mortality in patients with primary (ie, inheritable) or secondary immunosuppression (ie, related to hematological or solid cancers, autoimmune disorders, or organ transplants). In this framework, on August 12, 2022, PubMed and MEDLINE were searched for eligible studies published beginning with January 1, 2020—approximating the origins of the COVID-19 pandemic. Keywords and related Medical Subject Heading (MeSH) terms used in the search included: (*COVID-19* OR *SARS-CoV-2* OR *coronavirus disease 2019*) AND (*convalescent plasma* OR *immune plasma* OR *hyperimmune plasma*) AND (*immunosuppression* OR *immunodeficiency* OR *immunocompromised* OR *cancer* OR *transplant* OR *malignancy* OR *hematological* OR *oncologic* OR *lymphoma* OR *leukemia* OR *myeloma* OR *agammaglobulinemia* OR *hypogammaglobulinemia* OR *common variable immunodeficiency* OR *autoimmune disorder*). On August 12, 2022, nonsystematic searches of both Google Scholar and medRχiv were performed, which included abstracts of congress presentations that were not published yet. To be eligible for inclusion, full-text translations must have been available in English. References of included articles were examined for potential inclusion.

### Eligibility Criteria

Eligible patients had primary or secondary immunosuppression with a confirmed diagnosis of COVID-19. The intervention investigated was transfusion with COVID-19 convalescent plasma of any dosage. The control group was treated with standard of care according to local treatment guidelines, with or without a placebo. Eligible studies reported information on patients’ clinical outcomes after transfusion with COVID-19 convalescent plasma. To perform a comprehensive analysis, the retrieved literature was grouped into 3 different strata, according to information characteristics: (1) controlled trials underwent a quantitative analysis (meta-analysis); (2) large case series with aggregated data underwent a descriptive analysis; and (3) case reports and case series with individual patient data underwent a single patient analysis.

### Selected and Data Abstraction Processes

**Figure 1.**
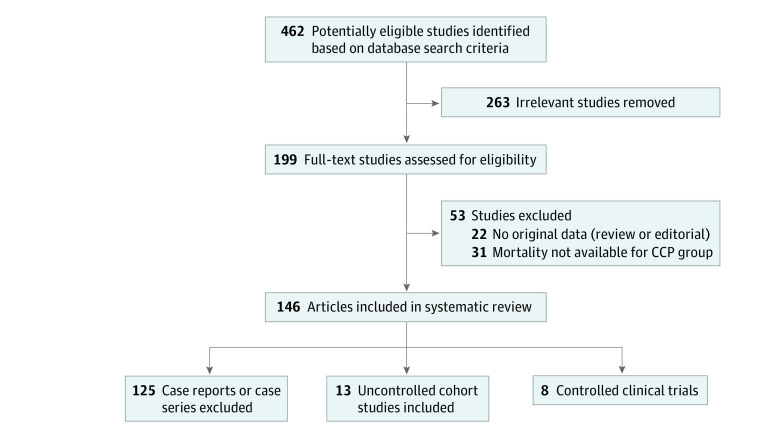
Flow Diagram of Study Inclusion CCP indicates COVID-19 convalescent plasma.

The data collection process was performed using a reciprocally blind evaluation by 2 reviewers (J.W.S. and M.F.), and disagreements were resolved by a third senior reviewer (D.F.). Further information on the selection process is presented in Figure 1. Data abstraction was performed using a standardized data abstraction form. Abstracted data, as available, included: patient’s sex and age, the underlying primary or secondary immunodeficiency, the 11-point WHO COVID-19 disease severity score,^22^ the need for mechanical ventilation, survival at the end of follow-up, the number of COVID-19 convalescent plasma units transfused, the volume of each COVID-19 convalescent plasma unit, the total COVID-19 convalescent plasma volume transfused, the antibody level (either neutralizing antibodies titer or anti-spike IgG levels) and the antibody test used, time from admission to COVID-19 convalescent plasma transfusion, time from symptom onset to COVID-19 convalescent plasma transfusion, rapid clinical improvement (defined as a reduction in supplemental oxygen requirements within 5 days of COVID-19 convalescent plasma transfusion), duration of follow-up (days), need for admission to intensive care unit (ICU), ICU length of stay (days; total and after COVID-19 convalescent plasma transfusion), concomitant COVID-19 antiviral treatments (intravenous immunoglobulins, remdesivir, hydroxychloroquine, anti-Spike monoclonal antibodies), and specific immunosuppressive drugs (anti–CD20 monoclonal antibodies).

### Study Risk of Bias Assessment

A risk of bias assessment was conducted using the Cochrane Risk of Bias 2.0 Tool for randomized clinical trials^23^ and the Risk Of Bias In Nonrandomized Studies of Interventions (ROBINS-I) for matched cohort studies.^24^ For both risk of bias assessment tools, each domain can score low risk if there is no indication for risk of bias, some concerns if there is potential for risk of bias, or high risk if there is clear indication for risk of bias. Two reviewers (M.F. and M.C.) independently applied the risk of bias assessment. Discrepancies were discussed until consensus.

### Statistical Analysis

#### Effect of Intervention

Measures of treatment effect were relative risk ratio (RR) and risk difference (RD). The study weight was calculated using the Mantel-Haenszel method. We assessed statistical heterogeneity using *t*^2^, Cochran’s Q, and *I^2^* statistics.^25^ The *I^2^* statistic describes the percentage of total variation across trials due to heterogeneity rather than sampling error. In the case of no heterogeneity (*I*^2^ = 0), studies were pooled using a fixed-effects model. Where values of *I*^2^ were greater than 0, a random-effects analysis was undertaken.

#### Summary of Findings Tables

For the outcome mortality, we used the principles of the GRADE system to assess the quality of the body of evidence associated with specific outcomes and constructed a summary of findings tables using REVMAN 5.4.^26,27^ These tables present key information concerning the certainty of the evidence, the magnitude of the effect sizes of the interventions examined, and the sum of available data for the main outcomes. The summary of findings tables also include an overall grading of the evidence related to each of the main outcomes using the GRADE approach, which defines the certainty of a body of evidence as the extent to which one can be confident that an estimate of effect or association is close to the true quantity of specific interest. The certainty of a body of evidence involves consideration of within-trial risk of bias (methodological quality), directness of evidence, heterogeneity, precision of effect estimates, and risk of publication bias.

#### Exploratory Analysis of Individual Patient Data

In the descriptive statistics of individual patient data, continuous variables were reported as mean (SD) or median (range) as appropriate according to distribution, while categorical variables were reported as numbers and percentages. Details associated with these analyses are provided in eTables 2 to 7 in the Supplement.

#### Exploratory Analysis of the Association Between COVID-19 Convalescent Plasma Volume and Mortality

In an exploratory analysis, we examined mortality rates stratified according to the transfused volume of COVID-19 convalescent plasma. For these analyses, COVID-19 convalescent plasma volume was stratified in 200 mL increments starting with a volume of less than or equal to 200 mL and ending with a volume of 1800 mL or more. Then, a breakpoint at 600 mL of COVID-19 convalescent plasma was tentatively posed, comparing the mortality when the COVID-19 convalescent plasma was under or over the level by Fisher exact test.

The basic model consisted in a logistic regression using mortality as the dependent variable and total volume as the estimator. The COVID-19 convalescent plasma total volume was expressed in units of 100 mL, for ease of interpretation. The potential additive independent effects of putative confounding variables, including: age, sex, time from admission to transfusion, rapid improvement of COVID-19 (within 5 days), ICU length of stay, and use of concomitant therapies (steroids, remdesivir, hydroxychloroquine, antibiotics, and anti–CD20 monoclonal antibodies), and immunosuppressive condition were evaluated.

#### Power Analysis

In power analysis, the total sample size was calculated to detect an experimental-group proportion of 0.06 as the death rate, with the control-group proportion of 0.08, assuming a 1-sided hypothesis test with a 5% significance level, focusing a desired power of 80%, and if both groups (treated and untreated) had the same number of observations. This would correspond to the prevention of 25% of the basal deaths or a risk ratio (RR) of 0.75. Stata version 17.0 (StataCorp) was used for all statistical calculations. Statistical analysis took place from July to November 2022.

## Results

### Study Selection and Characteristics

The process of study selection is represented in the PRISMA flow diagram (Figure 1). Three randomized clinical trials (RCTs)^28,29,30^ enrolling 214 participants and 5 matched cohort studies^31,32,33,34,35^ enrolling 1560 participants were included in the meta-analysis. Descriptive and exploratory analyses were performed on uncontrolled studies. For these exploratory analyses, 13 uncontrolled large case series without individual patient data enrolling 358 participants were included in descriptive analysis.^36,37,38,39,40,41,42,43,44,45,46,47,48^ In this study, 125 case reports or case series enrolling 265 participants^42,49,50,51,52,53,54,55,56,57,58,59,60,61,62,63,64,65,66,67,68,69,70,71,72,73,74,75,76,77,78,79,80,81,82,83,84,85,86,87,88,89,90,91,92,93,94,95,96,97,98,99,100,101,102,103,104,105,106,107,108,109,110,111,112,113,114,115,116,117,118,119,120,121,122,123,124,125,126,127,128,129,130,131,132,133,134,135,136,137,138,139,140,141,142,143,144,145,146,147,148,149,150,151,152,153,154,155,156,157,158,159,160,161,162,163,164,165,166,167,168,169,170,171^ were included for patient-level exploratory analyses. One study^42^ was included in both the descriptive analysis and the individual patient data analysis because individual patient data were available only for a subgroup of patients.

### Risk Assessment

The results of the risk of bias assessment for RCTs and matched cohort studies are presented in Table 1 and Table 2, respectively. One RCT was rated as good quality with low risk, whereas there was some concern with 1 RCT and 1 RCT had a high risk of bias. The greater risk of bias in 2 RCTs was associated with deviations from intended interventions, primarily owing to offering untreated patients to receive COVID-19 convalescent in the absence of clinical improvement. The matched cohort studies were judged at high risk of bias because they were open label trials. However, assessor masking has unclear importance for the outcome mortality because the risk of ascertainment bias is limited.

**Table 1.** Risk of Bias Among Randomized Clinical Trials

Trial	Risk of bias^a^					
	Randomization process	Deviations from intended interventions	Missing outcome data	Measurement of the outcome	Selection of the reported result	Overall
Bar et al,^28^ 2021	Low	Some concerns	Low	Low	Low	Some concerns
Lacombe et al,^30^ 2022	Low	Low	Low	Low	Low	Low
Denkinger et al,^29^ 2022	Low	High	Low	Low	Low	High

^a^ Risk of bias was assessed using the Cochrane Risk of Bias 2 tool.

**Table 2.** Risk of Bias Among Matched Cohort Studies

Trial	Risk of bias^a^						
	Confounding	Selection bias	Bias in measurement classification of interventions	Bias due to deviations from intended interventions	Bias due to missing data	Bias in measurement of outcomes	Bias in selection of the reported results
Biernat et al,^31^ 2021	High	High	High	Some concerns	Some concerns	Some concerns	Some concerns
Cristelli et al,^32^ 2021	High	Low	Low	Low	Low	Low	Low
Hueso et al,^34^ 2022	High	Low	Low	Low	Low	Low	Low
Lanza et al,^35^ 2022	High	Some concerns	Low	Low	Some concerns	Low	Low
Thompson et al,^33^ 2021	High	High	Low	Some concerns	Some concerns	High	Low

^a^ Risk of bias was assessed using the Risk of Bias in Non-Randomized Studies of Interventions (ROBINS-I) for interventional studies.

### Association Between Convalescent Plasma Transfusion and Mortality in Hospitalized Patients With Primary or Secondary Immunosuppression and COVID-19

**Figure 2.**
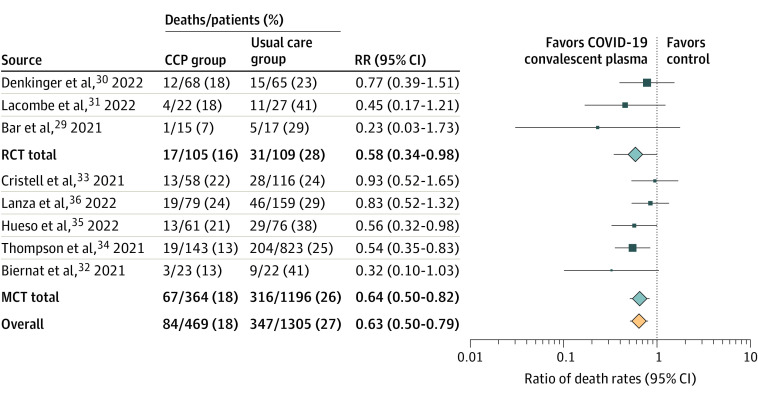
Forest Plot of Mortality Among Randomized Clinical Trials and Matched Cohort Studies Different size symbols indicate relative weights used in meta-analysis and are proportional to study size and study variance. Abbreviations: CCP, COVID-19 convalescent plasma; CI, confidence interval; MCT, matched cohort study; RCT, randomized clinical trial; RR, risk ratio.

In the primary meta-analysis of the 8 controlled trials (totaling 469 patients treated with COVID-19 convalescent plasma and 1305 controls),^28,29,30,31,32,33,34,35^ the key findings are summarized in Table 3, Figure 2, and eFigure 1 in the Supplement. There was a high level of concordance among study outcomes, and treatment with COVID-19 convalescent plasma was associated with reduced risk of mortality according to the pooled risk ratio of 0.63 (95% CI, 0.50 to 0.79) and the pooled risk difference of −0.10 (95% CI, −0.15 to −0.06).

**Table 3.** Summary of Findings for the 8 Controlled Studies Included in the Meta-analysis^**a**^

All cause mortality	Illustrative comparative risks (95% CI)^b^		Relative effect, RR (95% CI)	No. of participants	Quality of the evidence (GRADE)^c^	Comments
	Assumed risk, controls (standard of care)	Corresponding risk, intervention (convalescent plasma)				
All studies (RCTs and non-RCTs)	265 per 1000	172 per 1000 (from 132 to 207)	0.63 (0.50 to 0.78)	1774 Patients (8 trials, 3 RCTs and 5 non-RCTs)	2 of 4; Low (downgraded for serious ROB)	Mortality was observed more commonly among SOC recipients compared with CCP
RCTs only	284 per 1000	165 per 1000 (from 97 to 278)	0.58 (0.34/0.98)	214 Participants (3 RCTs)	3 of 4; Moderate (downgraded for ROB)	CCP reduces mortality compared to SOC
Cohort studies only	264 per 1000	169 per 1000 (from 132 to 216)	0.64 (0.50/0.82)	1560 Participants (5 trials)	2 of 4; Low (downgraded for serious risk of bias)	Mortality was observed more commonly among SOC recipients compared with CCP. In sensitivity analysis, exclusion of individual studies did not affect the effect size of intervention

Abbreviations: CCP, COVID-19 convalescent plasma; ROB, risk of bias; RR, risk ratio; SOC, standard of care.

^a^ The study included immunocompromised patients who were hospitalized with COVID-19 and treated with COVID-19 convalescent plasma. The comparison was the standard of care (SOC).

^b^ The basis for the assumed risk is the mean control group risk across studies. The corresponding risk (and its 95% CI) is based on the assumed risk in the comparison group and the relative effect of the intervention (and its 95% CI).

^c^ GRADE Working Group grades of evidence: (1) very low quality we are very uncertain about the estimate; (2) low quality, further research is very likely to have an important impact on our confidence in the estimate of effect and is likely to change the estimate; (3) moderate quality: further research is likely to have an important impact on our confidence in the estimate of effect and may change the estimate; and (4) high quality, further research is very unlikely to change our confidence in the estimate of effect.

### Exploratory Analyses of Individual-level Data

#### Participant Characteristics

The demographic and clinical characteristics of the individual patient data are summarized in eTable 3 in the Supplement. Among the 265 participants included in patient-level analyses the median (range) age was 55 (1-88) years, and 105 (40%) were females. Mean World Health Organization (WHO) disease severity score was 4.4, with 51 of 218 patients (23.4%) being in ICU on mechanical ventilation. The reported mortality rate was 31 of 265 patients (11.6%).

#### COVID-19 Convalescent Plasma Treatment

COVID-19 convalescent plasma treatment-related data associated with the individual-level patient data are summarized in eTable 4 in the Supplement. The mean (SD) number of COVID-19 convalescent plasma units transfused per patient was 2.3 (1.7), while the mean cumulative COVID-19 convalescent plasma volume transfused per patient was 460 ml (372 mL). Unfortunately, it was not possible to calculate the mean neutralizing antibody titer or to correlate the patients’ outcome with neutralizing antibody titers due to the wide heterogeneity of tests used (virus neutralization or high-throughput serology). No severe adverse reactions to COVID-19 convalescent plasma were reported. The median (range) time between symptom onset and COVID-19 convalescent plasma therapy was 17 (1 to 132) days, while the median (range) time between hospital admission and COVID-19 convalescent plasma therapy was 11 days (0 to 120). The median (range) follow-up period of the patients included in this single patients’ analysis was 19 (4 to 263 days; data available for 69 patients).

### Exploratory Analyses of COVID-19 Convalescent Plasma Volume and Mortality

Using individual-level data, mortality and COVID-19 convalescent plasma volume were described for 126 participants and these data are summarized in eFigure 2, eFigure 3, and eTable 5 in the Supplement. Seven death events were observed (ie, 6 males and 1 female) among the group of 92 patients where the COVID-19 convalescent plasma total volume did not exceed 600 mL. However, the comparison of the mortality when the COVID-19 convalescent plasma was under (7 events, 92 patients) or over this level (0 events, 34 patients), was not significant. The coefficients of the basic logistic model are reported in eTable 6 in the Supplement.

## Discussion

Several scientific societies (eg, ECIL-9,^172^ CDC/IDSA,^173^ and AABB^174^) have recently revised their guidelines to recommend the use of COVID-19 convalescent plasma in patients who are immunocompromised,^16,17^ especially after concerns related to the prevalence of monoclonal antibody-resistant SARS-CoV-2 variants. The hypothesis of a significant beneficial effect of COVID-19 convalescent plasma on mortality in patients who are immunocompromised cannot be definitively demonstrated with the present data, but very strong elements support its efficacy. The efficacy of antibody-based therapies for immunocompetent individuals is predicated on early administration with sufficient dosage.^175^ This principle was validated by the experience of COVID-19 convalescent plasma.^9^ While several immunocompromised cases have been treated with COVID-19 convalescent plasma derivatives (hyperimmune immunoglobulins),^176^ COVID-19 convalescent plasma is superior in turnaround times and inclusion of classes other than IgG.^177^ However, we note that the patients who are immunocompromised in this study were treated relatively late after the initial symptoms (17 days) and hospital admission (11 days) and yet our analysis suggests a benefit associated with COVID-19 convalescent plasma. For life-threatening COVID-19, the pathogenesis involves exuberant tissue-damaging inflammatory responses that follow an initial viral phase. Antibody-based therapies function primarily as antiviral agents and are much less likely to be affected in individuals who are in the inflammatory phase. However, individuals who are immunocompromised are generally unable to mount strong antibody or inflammatory responses and often cannot clear SARS-CoV-2. Hence, patients who are immunocompromised represent a biologically different population from the population that is not immunocompromised where antibody-based therapies may retain efficacy late into the course of disease.

The efficacy of COVID-19 convalescent plasma in patients who are immunocompromised and had reported symptoms for weeks or months paves the way to the hypothesis that COVID-19 convalescent plasma retains clinical efficacy until the recipient is seronegative and there is no irreversible parenchymal damage. The recently reopened COVID-19 convalescent plasma arm of the REMAP-CAP randomized controlled trial in UK will specifically target patients who are immunocompromised in the intensive care unit focusing on COVID-19 convalescent plasma from vaccinated donors (so-called Vax-Plasma or hybrid plasma).^178^ While most studies reported in this systematic review used COVID-19 convalescent plasma from unvaccinated donors (with a few exceptions^132,158^), it is noteworthy that Vax-Plasma is now widely available from regular donors and retains higher neutralizing antibody titers and efficacy against most SARS-CoV-2 variants.^179^

### Limitations

This study had limitations. First, our analyses included exploratory analysis of lower epistemological levels of evidence (ie, uncontrolled case series and reports). These data should not be used to infer definitive treatment effects but may provide relevant information describing the use of COVID-19 convalescent plasma under specific disease conditions. Second, we did not have access to patient-level data for many of the studies included in this article. This dearth of patient-level data does not allow analyses using more complex statistical models that incorporate multiple characteristics. Third, we limited our focus to a single outcome—all-cause mortality.

## Conclusions

This systematic review and meta-analysis suggests that convalescent plasma was associated with a mortality benefit among hospitalized patients with primary or secondary immunosuppression and COVID-19. Although these summary findings are encouraging for the use of therapeutic convalescent plasma in COVID-19 patients with primary or secondary immunosuppression, there remains a paucity of well-controlled, published data in these important patient populations. The clinical use of COVID-19 convalescent plasma and Vax-Plasma in patients who are immunocompromised and have COVID-19 may warrant further investigation.

## Notes

Supplement 1. **eFigure 1.** Forest Plot of Mortality Among Randomized Clinical Trials and Matched Cohort Studies **eFigure 2.** Association Between Convalescent Plasma Volume and Mortality Among Hospitalized Patients With Primary or Secondary Immunosuppression and COVID-19 **eFigure 3.** Association Between Convalescent Plasma Volume and Predicted Probability of Death Among Hospitalized Patients With Primary or Secondary Immunosuppression and COVID-19 **eTable 1.** PRISMA 2020 Checklist **eTable 2.** Summary of Uncontrolled Studies With Aggregated Results on the CCP Use in Immunocompromised Patients **eTable 3.** Characteristics of Patients Included in Individual Patient Analysis **eTable 4.** COVID-19 Convalescent Plasma Treatment-Related Data Among 265 Individual Patients **eTable 5.** Mortality Stratified by COVID-19 Convalescent Plasma Volume **eTable 6.** Logistic Regression Table of Coefficients **eTable 7.** Patient-Level Data

Supplement 2.
Data-Sharing Statement
