# Supplementary material for: COVID-19 Convalescent Plasma for the Treatment of Immunocompromised Patients: A Systematic Review and Meta-analysis
Source: JAMA Netw Open. 2023 Jan 12;6(1):e2250647. doi: 10.1001/jamanetworkopen.2022.50647 (PMC9857047; doi:10.1001/jamanetworkopen.2022.50647)
Supplement: Supplement 1. — eFigure 1. Forest Plot of Mortality Among Randomized Clinical Trials and Matched Cohort Studies eFigure 2. Association Between Convalescent Plasma Volume and Mortality Among Hospitalized Patients With Primary or Secondary Immunosuppression and COVID-19 eFigure 3. Association Between Convalescent Plasma Volume and Predicted Probability of Death Among Hospitalized Patients With Primary or Secondary Immunosuppression and COVID-19 eTable 1. PRISMA 2020 Checklist eTable 2. Summary of Uncontrolled Studies With Aggregated Results on the CCP Use in Immunocompromised Patients eTable 3. Characteristics of Patients Included in Individual Patient Analysis eTable 4. COVID-19 Convalescent Plasma Treatment-Related Data Among 265 Individual Patients eTable 5. Mortality Stratified by COVID-19 Convalescent Plasma Volume eTable 6. Logistic Regression Table of Coefficients eTable 7. Patient-Level Data [file jamanetwopen-e2250647-s001.pdf]

Supplemental Online Content

Senefeld JW, Franchini M, Mengoli C, et al. COVID-19 convalescent plasma for the treatment of immunocompromised patients: a systematic review and meta-analysis. *JAMA Netw Open*. 2023;6(1):e2250647. doi:10.1001/jamanetworkopen.2022.50647

eFigure 1. Forest Plot of Mortality Among Randomized Clinical Trials and Matched Cohort Studies

eFigure 2. Association Between Convalescent Plasma Volume and Mortality Among Hospitalized Patients With Primary or Secondary Immunosuppression and COVID-19

eFigure 3. Association Between Convalescent Plasma Volume and Predicted Probability of Death Among Hospitalized Patients With Primary or Secondary Immunosuppression and COVID-19

eTable 1. PRISMA 2020 Checklist

eTable 2. Summary of Uncontrolled Studies With Aggregated Results on the CCP Use in Immunocompromised Patients

eTable 3. Characteristics of Patients Included in Individual Patient Analysis

eTable 4. COVID-19 Convalescent Plasma Treatment-Related Data Among 265 Individual Patients

eTable 5. Mortality Stratified by COVID-19 Convalescent Plasma Volume

eTable 6. Logistic Regression Table of Coefficients

eTable 7. Patient-Level Data

This supplemental material has been provided by the authors to give readers additional information about their work.

© 2023 Senefeld JW et al. *JAMA Network Open.*

eFigure 1. Forest Plot of Mortality Among Randomized Clinical Trials and Matched Cohort Studies

Source	Deaths/patients (%) CCP Group	Deaths/patients (%) Usual care group	Risk Difference (95% CI)
Denkinger et al, 2022	12/68 (18%)	15/65 (23%)	-0.05 (-0.19 to 0.08)
Lacombe et al, 2022	4/22 (18%)	11/27 (41%)	-0.23 (-0.47 to 0.02)
Bar et al, 2021	1/15 (7%)	5/17 (29%)	-0.23 (-0.48 to 0.02)
**RCT Total**	17/105 (16%)	31/109 (28%)	-0.12 (-0.23 to -0.01)
Cristelli et al, 2021	13/58 (22%)	28/116 (24%)	-0.02 (-0.15 to 0.12)
Lanza et al, 2022	19/79 (24%)	46/159 (29%)	-0.05 (-0.17 to 0.07)
Thompson et al, 2021	19/143 (13%)	204/823 (25%)	-0.12 (-0.18 to -0.05)
Hueso et al, 2022	13/61 (21%)	29/76 (38%)	-0.17 (-0.32 to -0.02)
Biernat et al, 2021	3/23 (13%)	9/22 (41%)	-0.28 (-0.53 to -0.03)
**MCT Total**	67/364 (18%)	316/1,196 (26%)	-0.10 (-0.15 to -0.05)
**Overall**	84/469 (18%)	347/1,305 (27%)	-0.10 (-0.15 to -0.06)

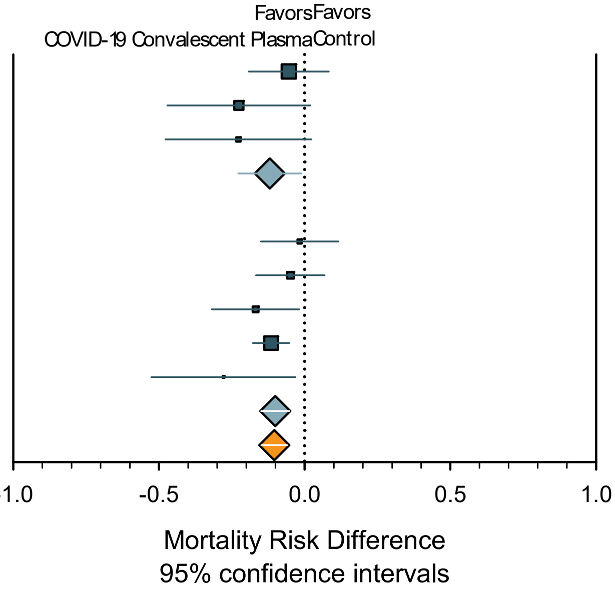

Different size symbols indicate relative weights used in meta-analysis and are proportional to study size and study variance. Abbreviations: CCP, COVID-19 convalescent plasma; CI, confidence interval; RCT, randomized clinical trial; MCT, matched cohort study.

© 2023 Senefeld JW et al. *JAMA Network Open.*

eFigure 2. Association Between Convalescent Plasma Volume and Mortality Among Hospitalized Patients With Primary or Secondary Immunosuppression and COVID-19

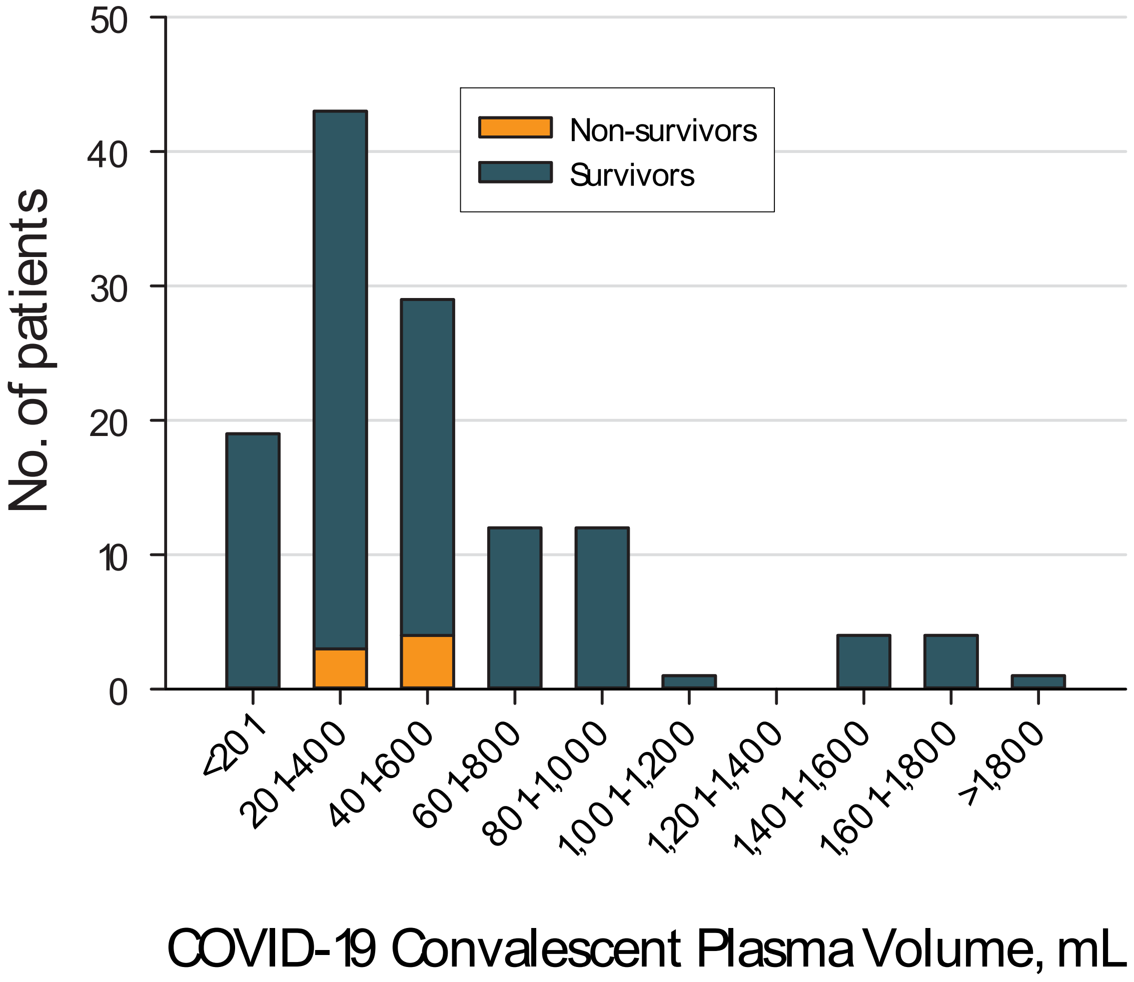

Stacked bar chart displaying the number of patients transfused with different volumes of COVID-19 convalescent plasma. Orange bars represent number of non-survivors, blue bars represent number of patients at risk (survivors), and the height of the stacked bar represents the total number of patients within each strata of COVID-19 convalescent plasma volume.

© 2023 Senefeld JW et al. *JAMA Network Open.*

eFigure 3. Association Between Convalescent Plasma Volume and Predicted Probability of Death Among Hospitalized Patients With Primary or Secondary Immunosuppression and COVID-19

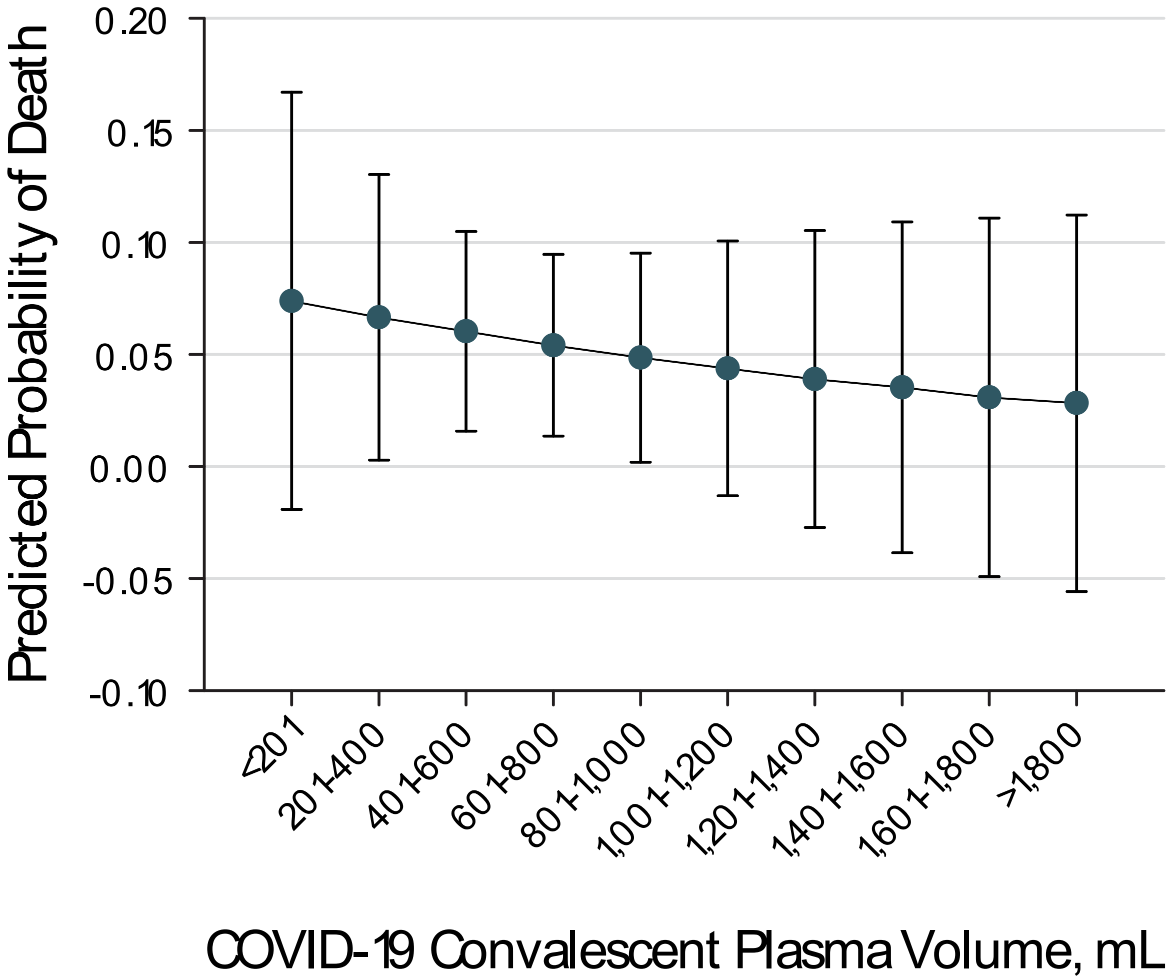

Line and scatter plots displaying the predicted probability of death stratified by volume of COVID-19 convalescent plasma derived using logistic regression, basic model. The large confidence intervals highlight the dearth of patient level data, suggesting the need for additional data describing mortality among hospitalized patients with primary or secondary immunosuppression and COVID-19 treated with convalescent plasma. The data underlying this predictive model are represented in **eFigure 2** and **eTable 5**, and represent 125 patients transfused with COVID-19 convalescent plasma.

© 2023 Senefeld JW et al. *JAMA Network Open.*

eTable 1. PRISMA 2020 Checklist

Section and Topic	Item #	Checklist item	Location where item is reported
**TITLE**			
Title	1	Identify the report as a systematic review.	Title page
**ABSTRACT**			
Abstract	2	See the PRISMA 2020 for Abstracts checklist.	Structured Abstract
**INTRODUCTION**			
Rationale	3	Describe the rationale for the review in the context of existing knowledge.	Introduction
Objectives	4	Provide an explicit statement of the objective(s) or question(s) the review addresses.	Introduction
**METHODS**			
Eligibility criteria	5	Specify the inclusion and exclusion criteria for the review and how studies were grouped for the syntheses.	Search Criteria
Information sources	6	Specify all databases, registers, websites, organisations, reference lists and other sources searched or consulted to identify studies. Specify the date when each source was last searched or consulted.	Search Criteria
Search strategy	7	Present the full search strategies for all databases, registers and websites, including any filters and limits used.	Search criteria
Selection process	8	Specify the methods used to decide whether a study met the inclusion criteria of the review, including how many reviewers screened each record and each report retrieved, whether they worked independently, and if applicable, details of automation tools used in the process.	Search Criteria
Data collection process	9	Specify the methods used to collect data from reports, including how many reviewers collected data from each report, whether they worked independently, any processes for obtaining or confirming data from study investigators, and if applicable, details of automation tools used in the process.	Search Criteria
Data items	10a	List and define all outcomes for which data were sought. Specify whether all results that were compatible with each outcome domain in each study were sought (e.g. for all measures, time points, analyses), and if not, the methods used to decide which results to collect.	Search Criteria
	10b	List and define all other variables for which data were sought (e.g. participant and intervention characteristics, funding sources). Describe any assumptions made about any missing or unclear information.	Search Criteria
Study risk of bias assessment	11	Specify the methods used to assess risk of bias in the included studies, including details of the tool(s) used, how many reviewers assessed each study and whether they worked independently, and if applicable, details of automation tools used in the process.	Systematic review of comparative studies
Effect measures	12	Specify for each outcome the effect measure(s) (e.g. risk ratio, mean difference) used in the synthesis or presentation of results.	Effect of Intervention
Synthesis methods	13a	Describe the processes used to decide which studies were eligible for each synthesis (e.g. tabulating the study intervention characteristics and comparing against the planned groups for each synthesis (item #5)).	Search Criteria
	13b	Describe any methods required to prepare the data for presentation or synthesis, such as handling of missing summary statistics, or data conversions.	Search Criteria
	13c	Describe any methods used to tabulate or visually display results of individual studies and syntheses.	'Summary of findings' tables
	13d	Describe any methods used to synthesize results and provide a rationale for the choice(s). If meta-analysis was performed, describe the model(s), method(s) to identify the presence and extent of statistical heterogeneity, and software package(s) used.	Effect of Intervention
	13e	Describe any methods used to explore possible causes of heterogeneity among study results (e.g. subgroup analysis, meta-regression).	Effect of Intervention

© 2023 Senefeld JW et al. *JAMA Network Open.*

Section and Topic	Item #	Checklist item	Location where item is reported
	13f	Describe any sensitivity analyses conducted to assess robustness of the synthesized results.	Effect of Intervention
Reporting bias assessment	14	Describe any methods used to assess risk of bias due to missing results in a synthesis (arising from reporting biases).	Systematic review of comparative studies
Certainty assessment	15	Describe any methods used to assess certainty (or confidence) in the body of evidence for an outcome.	'Summary of findings' tables
**RESULTS**			
Study selection	16a	Describe the results of the search and selection process, from the number of records identified in the search to the number of studies included in the review, ideally using a flow diagram.	Figure 1
	16b	Cite studies that might appear to meet the inclusion criteria, but which were excluded, and explain why they were excluded.	Figure 1
Study characteristics	17	Cite each included study and present its characteristics.	Table 1; Table 2; Table 3
Risk of bias in studies	18	Present assessments of risk of bias for each included study.	Figure 2
Results of individual studies	19	For all outcomes, present, for each study: (a) summary statistics for each group (where appropriate) and (b) an effect estimate and its precision (e.g. confidence/credible interval), ideally using structured tables or plots.	Table 1
Results of syntheses	20a	For each synthesis, briefly summarise the characteristics and risk of bias among contributing studies.	Figure 2
	20b	Present results of all statistical syntheses conducted. If meta-analysis was done, present for each the summary estimate and its precision (e.g. confidence/credible interval) and measures of statistical heterogeneity. If comparing groups, describe the direction of the effect.	Table 1
	20c	Present results of all investigations of possible causes of heterogeneity among study results.	Figure 3
	20d	Present results of all sensitivity analyses conducted to assess the robustness of the synthesized results.	Figure 3
Reporting biases	21	Present assessments of risk of bias due to missing results (arising from reporting biases) for each synthesis assessed.	Figure 2
Certainty of evidence	22	Present assessments of certainty (or confidence) in the body of evidence for each outcome assessed.	Table 1
**DISCUSSION**			
Discussion	23a	Provide a general interpretation of the results in the context of other evidence.	Discussion
	23b	Discuss any limitations of the evidence included in the review.	Discussion
	23c	Discuss any limitations of the review processes used.	Discussion
	23d	Discuss implications of the results for practice, policy, and future research.	Discussion
**OTHER INFORMATION**			
Registration and protocol	24a	Provide registration information for the review, including register name and registration number, or state that the review was not registered.	CRD42022316321
	24b	Indicate where the review protocol can be accessed, or state that a protocol was not prepared.	PROSPERO
	24c	Describe and explain any amendments to information provided at registration or in the protocol.	N/A
Support	25	Describe sources of financial or non-financial support for the review, and the role of the funders or sponsors in the review.	Article Information
Competing interests	26	Declare any competing interests of review authors.	Article Information
Availability of data, code and	27	Report which of the following are publicly available and where they can be found: template data collection forms; data extracted from	Article information

© 2023 Senefeld JW et al. *JAMA Network Open.*

Section and Topic	Item #	Checklist item	Location where item is reported
other materials		included studies; data used for all analyses; analytic code; any other materials used in the review.	

From: Page MJ, McKenzie JE, Bossuyt PM, Boutron I, Hoffmann TC, Mulrow CD, et al. The PRISMA 2020 statement: an updated guideline for reporting systematic reviews. BMJ 2021;372:n71. doi: 10.1136/bmj.n71

For more information, visit: http://www.prisma-statement.org/

© 2023 Senefeld JW et al. *JAMA Network Open.*

eTable 2. Summary of Uncontrolled Studies With Aggregated Results on the CCP Use in Immunocompromised Patients

First author, reference	Population	Diagnosis	COVID-19	CCP regimen	Days between symptoms and CCP	Previous or concomitant therapies	Outcome	Notes & Conclusions
Betrains [39]	5 F, median age 37 y (range 19-67 y)	5 NHL	Severe COVID-19	4 high-titer (≥1:160 VNT) CCP units	N/A	1 steroids, 3 HCQ, 1 antibiotics, 2 remdesivir	Increase in nAb titer following CCP<br>Overall mortality rate 1/5 (20%)	Patients with B-cell-depleted lymphomas are ideal candidates for CCP therapy
Gharbharan [37]	25 (15 M, 10 F), median age 53 y (IQR 44-66 y)	12 NHL<br>1 AL<br>8 AID<br>2 CLL<br>1 MS<br>1 AGG	19 severe COVID-19,<br>6 mild-moderate COVID-19	21 patients 1 CCP unit, 4 patients 2 CCP units	Median 26 d (IQR 15-34 d)	N/A	Overall mortality rate 4/25 (16%)	24/25 anti-CD20 therapy. The CCP benefit in B-cell depleted patients is present regardless of symptom duration.
Greenbaum[38]	44 (M 25, 19 F), median age 60 (range 37-48 y)	17 SC<br>27 HM	Severe COVID-19	42 patients 1 CCP unit, 2 patients 2 CCP units	N/A	32 remdesivir, 20 tocilizumab, 11 steroids, 1 anakinra	Overall mortality 12/44 (27.3%)	Shorter time from COVID-19 diagnosis to CCP administration (≤3 days) was associated with better survival
Hueso[49]	17 (12 M, 5 F), median age 58 y (range 35-77 y)	11 NHL<br>3 CLL<br>1 WM<br>1 CVID<br>1 MS	WHO score 4-7	CCP units ≥1:40 (VNT) or > 5.6 (ELISA)	Median 56 d (range 7-83 d)	8 steroids, 5 HCQ, 4 tocilizumab, 3 remdesivir, 2 antivirals	Rapid viral clearance following CCP Overall mortality rate 1/15 (6.7%)	15/17 previous treatment with anti-CD20 therapy.<br>CCP is a promising therapy for COVID-19 B-cell depleted patients
Jasuja[40]	22 (N/A)	22 KTR	N/A	N/A	N/A	HCQ, steroids, antibiotics, remdesivir, tocilizumab	Overall mortality rate 7/22 (31.8%)	CCP was associated with no clinical benefit in KTR
Jeyaraman[41]	33 (23 M, 10 F), median age 62 y (range 7-80 y)	18 NHL<br>4 AL<br>7 MM<br>2 MPD<br>2 MDS	Severe COVID-19	CCP units >1:640<br>18 patients 1 CCP unit, 15 patients 2 CCP units	Median 4 d (range 2-25 d)	HCQ, steroids, antibiotics, remdesivir, tocilizumab	Overall 42.day mortality rate 15/33 (45.5%).<br>No mortality difference between early (<7 days) versus late CCP transfusion	Study with methodological limitations (retrospective case series)
Levy[42]	50 (N/A)	23 NHL<br>9 MM<br>8 CLL<br>1 MPD<br>6 AL<br>2 MDS<br>1 HCL	40 severe/critical COVID-19; 10 mild/moderate COVID-19	N/A	N/A	Steroids, remdesivir	Overall mortality rate 16/50 (32%)	CCP was associated with no clinical benefit
Ljunquist[43]	28 (13 M, 15 F), median age 56 (range 16-84 y)	13 HM<br>5 SOT<br>2 PI	Severe COVID-19	76 CCP units transfused (median nAb titer 1:141). 21 patients received 3 CCP units	Median 26 d (range 6-68 d)	23 steroids, 18 remdesivir	Overall 30 days mortality rate 6/28 (21.4%)	50% (14/28) of patients received rituximab.<br>Data not conclusive.

© 2023 Senefeld JW et al. *JAMA Network Open.*

First author, reference	Population	Diagnosis	COVID-19	CCP regimen	Days between symptoms and CCP	Previous or concomitant therapies	Outcome	Notes & Conclusions
Magyari[44]	20 (13 M, 7 F), median age 56 y (range 27-76 y)	10 NHL,	18 moderate COVID-19 (WHO score 4-5)	Median 4 CCP units (range 1-15 units)	Median 13.5 d (range 3-44 d)	17 patients received concomitant remdesivir, 20 steroids, 9 antivirals	No COVID-19 related deaths were recorded	Anti-CD20 therapy in 13/20 patients (65.0%). Clinical benefit of early combined administration of remdesivir and CCP
		1 MM	2 severe					
		4 CLL	COVID-19 (WHO score 8-9)					
		4 AL						
		1 MPD						
Tremblay[45]	24 (14 M, 10 F), median age 69 y (range 31-88 y)	5 NHL	Severe COVID-19	High titer (≥1:320) CCP units	N/A	16 HCQ, 15 antibiotics, 2 remdesivir, 1 tocilizumab	Overall mortality rate 41.2% (10/24)	Clinical benefit of CCP when administered early in the COVID-19 disease course
		4 MM						
		2 AL						
		1 HL						
		1 MPD						
		1 CLL						
		10 SC						
Rodionov[46]	14 (7 M, 7 F), median age 65 y (IQR 58-70 y)	8 SOT	Median WHO score 5 (range 4-6)	Titer ≥1:40 CCP units (VNT)	N/A	N/A	Overall mortality 2/14 (14.3%).	Immunosuppressed patients are candidates for CCP treatment
		4 HSCT		1 patient 1 CCP unit, 2 patients 2 CCP units, 11 patients 3 CCP units			8/14 (57.0%) showed clinical improvement on day 5 after CCP	
		2 HM						
Sait[47]	44 (N/A)	SOT	Median WHO score 4 (IQR 3-5)	N/A	N/A	Steroids, remdesivir, antibiotics	Overall mortality 3/44 (6.8%)	The use of CC is encouraged in SOT inpatients
Weinbergerova[48]	32 (19 M, 13 F), median age 57.7 y (range 25-86 y)	10 NHL	16/32 (50.0%) severe COVID-19	2 units high titer (≥1:160) CCP units	N/A	Steroids, remdesivir	3/32 (9.4%)	Early COVID-19 treatment with remdesivir + high titer CCP is effective in hematological patients
		10 AL						
		6 MM						
		3 CLL						
		2 MPD						
		1 AID						

Abbreviations: AL, acute leukemia; AID, autoimmune disorder; AGG, agammaglobulinemia; CCP, COVID-19 convalescent plasma; CLL, chronic lymphocytic leukemia; CVID, common variable immune deficiency; d, days; ELISA, enzyme-linked immunosorbent assay; F, females; HCL, hairy cell leukemia; HCQ, hydroxychloroquine; HL, Hodgkin's lymphoma; HM, hematological malignancy; HSCT, hematopoietic stem cell transplantation; IQR, interquartile range; KTR, kidney transplant recipients; M, males; MDS, myelodysplastic syndrome; MM, multiple myeloma; MPD, myeloproliferative disorders; MS, multiple sclerosis; N/A, not available; nAb, neutralizing antibody; NHL, non-Hodgkin's lymphoma; PI, primary immunodeficiency; SC, solid cancers; SOT, solid organ transplant; VNT, viral neutralization test; WHO, World Health Organization; WM, Waldenstrom macroglobulinemia; y, years.

© 2023 Senefeld JW et al. *JAMA Network Open.*

eTable 3. Characteristics of Patients Included in Individual Patient Analysis

**Characteristics (N = 265)**	**Data**
Age, median (range), y	55 (1-88)
Females/males, n	105/161
Mortality, n (%)	31/265 (11.6%)
**COVID-19 severity**	
WHO disease severity score, mean ± SD[1]	4.4 ± 1.8
Mechanical ventilation, n (%)	51/218 (23.4%)
ICU length of stay, days, median (range)[2]	33 (6-271)
**Immunosuppressive condition, n (%)**	
**Primary immunosuppression**	47/265 (17.7%)
Agammaglobulinemia	20/47 (42.6%)
Common variable immunodeficiency	22/47 (46.8%)
Others	5/47 (10.6%)
**Secondary immunosuppression**	219/265 (82.3%)
**Hematological malignancies**	134/219 (61.2%)
Non-Hodgkin's lymphoma	75/134 (56.0%)
Chronic lymphocytic leukemia	17/134 (12.7%)
Multiple myeloma	5/134 (3.7%)
Myelodysplastic syndrome	2/134 (1.5%)
Acute leukemia	13/134 (9.7%)
Myeloproliferative disorders	3/134 (2.2%)
HSCT	4/134 (3.0%)
PHLH	1/134 (0.7%)
Not specified	14/134 (10.4%)
**Solid cancers**	6/219 (2.7%)
Sarcoma	1/6 (16.7%)
Wilm's tumor	1/6 (16.7%)

© 2023 Senefeld JW et al. *JAMA Network Open.*

Characteristics (N = 265)	Data
Thymoma	2/6 (33.2%)
Lung cancer	1/6 (16.7%)
Prostate cancer	1/6 (16.7%)
**Solid organ transplants**	65/219 (29.7%)
Kidney	43/65 (66.1%)
Liver	14/65 (21.5%)
Heart	7/65 (10.8%)
Not specified	1/65 (1.5%)
**Autoimmune disorders**	12/219 (4.5%)
Systemic lupus erythematosus	2/12 (16.7%)
Sjogren syndrome	1/12 (8.3%)
Rheumatoid arthritis	2/12 (16.7%)
MCVD	2/12 (16.7%)
Others	5/12 (41.7%)
**Infective**	2/219 (0.9%)
HIV	2/2 (100%)
**Concomitant therapies, n (%)**	
**COVID-19 specific treatments, n (%)**	
Remdesivir	111/265 (41.7%)
IVIG	45/265 (16.9%)
Hydroxychloroquine	40/265 (15.0%)
Steroids	142/265 (53.4%)
**Anti-SARS-CoV-2 monoclonal antibodies**	11/265 (4.1%)
Casirivimab + imdevimab	3/11 (27.3%)
Bamlanivimab	4/11 (36.4%)
Bamlanivimab + etesivimab	3/11 (27.3%)
Not specified	1/11 (9.0%)
Antibiotics	125/265 (47.0%)

© 2023 Senefeld JW et al. *JAMA Network Open.*

Characteristics (N = 265)	Data
Other therapeutics[3]	25/265 (9.4%)
**Active immunosuppressive treatment, n (%)**	
**Chemotherapy**	47/265 (17.7%)
Anti-CD20 monoclonal antibodies[4]	35/47 (74.5%)
CAR-T	3/47 (6.4%)
Others	9/47 (19.1%)
Other immunosuppressive agents[5]	11/265 (4.1%)

Abbreviations: CAR-T, Chimeric Antigen Receptor T-cell therapies; HIV, human immunodeficiency virus; HSCT, hematopoietic stem cell transplantation; ICU, intensive care unit; IVIG, intravenous immunoglobulin; LMWH, low molecular weight heparin; MCVD, mixed collagen vascular disorder; PHLH, primary hemophagocytic lymphohistiocytosis; SD, standard deviation; WHO, World Health Organization.

[1]Data available in 40 patients.

[2]Data available in 27 patients.

[3]Including protease inhibitors, antivirals, anti-IL-1 and anti-IL-6 drugs.

[4]Rituximab or obinutuzumab.

[5]Azathioprine, tacrolimus, methotrexate, mycophenolate, infliximab.

© 2023 Senefeld JW et al. *JAMA Network Open.*

**eTable 4.** COVID-19 Convalescent Plasma Treatment-Related Data Among 265 Individual Patients		
Parameters	Information available for no. patients	Results
CCP transfused units, mean ± SD (range)	153	2.3 ± +1.7 (1-11)
CCP total transfused volume, mL, mean ± SD (range)	126	460 ± 372 (200-1800)
Days between symptom onset and CCP therapy, median (range)	188	17 (1-132)
Days between hospital admission and CCP therapy, median (range)	101	11 (0-120)
Post-CCP rapid improvement in oxygen supplementation (≤ 5 days), n (%)	114	64 of 114 patients (56.1%)

Abbreviations: CCP, COVID-19 convalescent plasma; n, number of patients; SD, standard deviation

© 2023 Senefeld JW et al. *JAMA Network Open.*

eTable 5. Mortality Stratified by COVID-19 Convalescent Plasma Volume

COVID-19 Convalescent Plasma Volume, mL	Non-survivors, n	Survivors, n
<201	0	19
201 to 400	3	40
401 to 600	4	25
601 to 800	0	12
801 to 1,000	0	12
1,001 to 1,200	0	1
1,201 to 1,400	0	0
1,401 to 1,600	0	4
1,601 to 1,800	0	4
>1,800	0	1
Total	7	118

Association between convalescent plasma volume and mortality among hospitalized patients with primary or secondary immunosuppression and COVID-19. Seven death events (5.6%) were observed of 125 patients transfused with a defined volume of COVID-19 convalescent plasma.

© 2023 Senefeld JW et al. *JAMA Network Open.*

**eTable 6.** Logistic Regression Table of Coefficients					
Mortality	Coefficient	Standard Error	z	P	95% confidence interval
COVID-19 convalescent plasma volume	-0.0551	0.1156	-0.48	0.634	(-0.2818 to 0.1716)
_cons	-2.537	0.6998	-3.63	0	(-3.9086 to -1.1654)

Mortality was the dependent variable, CCP total volume was the predictor. The CCP total volume was expressed in units of 100 mL. Number of obs = 125, log likelihood = -26.896492, LR chi2(1) = 0.28, P = 0.5994, Pseudo R2 = 0.0051.

© 2023 Senefeld JW et al. *JAMA Network Open.*

eTable 7. Patient-Level Data (Part I)

No.	Study	patient no.	Sex	Age, y	Mechanical Ventilation?	Mortality?
1	Abid	1	M	76	Yes	Yes
		2	M	88	No	No
2	Adedoyin O	1	M	70	Yes	Yes
		2	F	69	No	No
3	Antony	1	M	63	Yes	No
4	Avanzato	1	F	71	No	No
5	Baang	1	M	60	No	No
6	Bakhsh A	1	F	54	Yes	No
7	Balashov	1	F	1	No	No
8	Basheer M	1	M	N/A	N/A	No
9	Bayrak	1	M	50	No	No
10	Belcari	1	M	62	No	No
11	Bosnjak B	1	F	N/A	No	No
12	Bronstein	1	M	68	No	No
13	Bruiners N	1	F	50	Yes	No
14	Buckland	1	M	31	N/A	No
15	Casarola G	1	F	53	No	No
16	Chen L	1	M	55	No	Yes
17	Choudhury A	1	M	52	No	No
18	Christensen J	1	F	64	Yes	Yes
19	Cinar O	1	M	55	No	No
20	Clark	1	F	76	No	No
21	Colombo	1	F	67	No	NO
22	Cusi MG	1	M	55	No	No
		2	F	77	No	No
		3	M	60	No	No
23	D'abramo	1	M	39	NO	NO
		2	M	66	YES	NO
		3	M	65	YES	NO
		4	M	67	YES	NO
		5	M	59	Yes	NO
		6	F	77	YES	NO
		7	M	54	NO	NO
24	Dale M	1	F	65	Yes	No
		2	F	58	Yes	No
25	Delgado-Fernandez	1	M	26	No	No
		2	M	66	No	No
		3	M	62	No	No
26	Dell'Isola	1	F	6	No	No
27	Deveci B	1	F	34	No	No
		2	F	45	No	No

© 2023 Senefeld JW et al. *JAMA Network Open.*

No.	Study	patient no.	Sex	Age, y	Mechanical Ventilation?	Mortality?
		3	M	66	No	No
28	Di Palma	1	M	40	no	no
29	Erber J	2	F	50-54	Yes	No
30	Ferrari	1	F	48	No	No
		2	M	60	No	No
		3	M	60	No	No
		4	M	43	No	No
		5	M	70	No	No
		6	M	69	No	No
		7	M	60	No	No
31	Franchini	1	M	60	No	No
		2	M	72	No	No
		3	M	78	No	Yes
		4	M	77	No	Yes
		5	M	72	No	No
		6	M	50	No	No
		7	F	74	No	No
		8	M	76	No	No
		9	M	6	Yes	No
32	Fung	1	M	44	Yes	No
		2	M	44	No	No
		3	F	65	No	No
		4	M	62	No	No
33	Furlan	1	M	53	No	No
		2	M	50	No	No
		3	M	49	No	No
		4	M	53	No	No
34	Gattuso G	1	M	11	No	No
35	Gordon O	1	M	9	No	No
		2	F	4	No	No
		3	M	16	No	No
		4	F	15	No	No
		5	M	17	No	No
		6	F	6	No	No
		7	F	5	No	No
		8	M	6	No	No
		9	M	9	No	No
36	Gupta	1	F	37	No	No
		2	M	49	No	No
		3	F	32	No	No
		4	M	49	No	No
		5	M	26	No	No
		6	M	52	No	No

© 2023 Senefeld JW et al. *JAMA Network Open.*

No.	Study	patient no.	Sex	Age, y	Mechanical Ventilation?	Mortality?
		7	M	51	Yes	Yes
		8	M	56	No	No
		9	M	30	No	No
		10	M	40	No	No
37	Halfmann PJ	1	M	50	Yes	No
38	Hanssen JLJ	1	M	81	No	No
39	Hartman (Prophylaxis)	1	M	35	No	No
40	Hatzl	1	M	52	N/A	No
		2	M	55	N/A	No
41	Ho	1	F	65	N/A	No
		2	F	39	N/A	Yes
		3	M	40	N/A	No
		4	M	24	N/A	No
		5	M	10	N/A	No
42	Honjo	1	F	72	No	No
43	Hovey	1	M	26	No	No
44	Hughes CM	1	M	53	No	No
45	Iaboni	1	M	28	No	No
46	Jamir	1	M	49	No	No
47	Jassem J	1	M	66	No	No
48	Jiang J	1	F	70	No	No
49	Jin H	1	M	10	No	No
		2	M	24	No	No
		3	M	40	No	No
50	Karaolidou F	1	M	45	No	No
51	Karatas	1	M	61	No	No
52	Katz-Greenberg	1	F	61	No	No
		2	M	45	No	No
		3	F	42	Yes	No
		4	F	59	Yes	No
53	Keitel	1	F	25	No	No
54	Kemp	1	M	75	N/A	Yes
55	Kenig	1	F	62	N/A	No
		2	F	47	N/A	No
		3	F	58	N/A	No
		4	M	63	N/A	No
		5	F	67	N/A	No
56	Ketels	1	M	38	Yes	No
57	Khan AM	1	M	41	No	No
58	Khatamzas	1	F	70	N/A	N/A
59	Khatri	1	M	68	Yes	Yes
60	Kluger	1	M	69	N/A	No

© 2023 Senefeld JW et al. *JAMA Network Open.*

No.	Study	patient no.	Sex	Age, y	Mechanical Ventilation?	Mortality?
		2	M	47	N/A	No
		3	M	50	N/A	No
61	Kremer AE	1	M	18	N/A	No
		2	M	70	N/A	No
		3	M	49	N/A	No
62	Kutzler	1	M	55	N/A	No
		2	F	70	N/A	Yes
63	Lancman	1	F	55	No	No
64	Lang-Meli J	1	M	61	N/A	No
		2	F	55	N/A	No
		3	M	71	N/A	No
		4	F	30	N/A	No
		5	F	53	N/A	No
		6	F	48	N/A	No
		7	F	66	N/A	No
		8	M	41	N/A	No
		9	M	25	N/A	No
		10	M	11	N/A	No
		11	M	13	N/A	No
		12	M	57	N/A	No
		14	F	57	N/A	No
		15	F	55	N/A	No
		16	M	60	N/A	No
65	Lazzari L	1	M	45	No	No
66	Lemus HN	1	F	54	NO	NO
67	Lima	1	M	62	Yes	no
		2	M	68	Yes	no
68	Lindemann 2021a (J Med Virol)	1	F	63	No	No
		2	F	62	No	No
69	Ljungquist O	1	M	52	No	No
		2	M	59	Yes	Yes
		3	M	64	No	No
		4	M	52	No	No
		5	F	55	No	No
		6	M	71	Yes	Yes
		7	M	68	No	Yes
		8	F	16	Yes	No
		9	F	78	No	No
		10	M	77	No	No
		11	M	74	No	No
		12	M	57	No	Yes
		13	M	61	Yes	Yes

© 2023 Senefeld JW et al. *JAMA Network Open.*

No.	Study	patient no.	Sex	Age, y	Mechanical Ventilation?	Mortality?
70	London	1	F	41	No	No
71	Lubnow	1	F	21	No	No
72	Luetkens	1	F	72	No	No
73	Madariaga	7	M	57	No	No
		8	M	30	No	No
74	Malsy	1	F	53	No	NO
75	Martens	1	M	60	No	No
76	Martínez-Barranco P	1	F	47	no	no
		2	M	76	no	yes
		3	F	82	no	no
		4	F	75	no	no
77	Martinez-Chincilla	1	M	47	No	No
		2	F	51	No	No
78	Martinot	1	F	76	No	No
79	McKemey	1	F	57	No	No
80	Mehta	1	M	50	Yes	Yes
		2	M	65	Yes	No
81	Mendes-Correa	1	M	45	No	No
82	Meyts	1	F	40	N/A	Yes
		2	M	40	Yes	No
		3	M	18	N/A	No
		4	M	12	N/A	No
		5	M	34	N/A	No
		6	M	74	N/A	No
83	Milosevic	1	M	34	No	No
84	Mira	1	M	39	No	No
85	Mohseni	1	F	53	No	No
86	Monrad	1	M	75	No	No
87	Moore	1	F	63	No	No
88	Moutinho-Pereira	1	F	39	No	No
89	Naeem	1	F	65	No	No
		2	F	35	No	No
		3	M	36	No	No
90	Nguyen	1	M	24	No	No
91	Niu	1	F	53	No	No
92	Nussenblatt	1	F	40	N/A	No
93	Nyström	1	M	30	No	No
94	Oliva	1	F	56	Yes	No
		2	F	68	Yes	No
		3	F	50	Yes	No
		4	F	63	Yes	No
		5	F	73	Yes	Yes

© 2023 Senefeld JW et al. *JAMA Network Open.*

No.	Study	patient no.	Sex	Age, y	Mechanical Ventilation?	Mortality?
		6	F	50	Yes	No
95	Ordaya EE	1	M	68	No	No
96	Ormazabal Velez	1	F	71	no	no
		2	M	60	no	no
97	Pal	1	F	70	No	No
		2	M	30	No	No
		3	F	53	No	No
		4	M	64	No	No
		5	F	68	No	No
		6	M	24	No	No
98	Pommeret F	1	F	34	No	Yes
		2	F	62	Yes	No
		3	F	63	No	No
		4	M	57	No	No
		5	F	67	NO	No
99	Prasad	1	F	73	Yes	No
100	Rahman	1	M	N/A	No	No
		2	M	N/A	No	No
		3	M	N/A	Yes	No
		4	M	N/A	No	Yes
		5	M	N/A	Yes	Yes
		6	M	N/A	No	No
		7	M	N/A	Yes	Yes
		8	M	N/A	No	No
		9	F	N/A	Yes	No
		10	F	N/A	No	No
		11	F	N/A	No	No
		12	F	N/A	Yes	No
		13	F	N/A	No	No
101	Reuken	1	F	56	Yes	No
102	Rem	1	F	72	YES	Yes
103	Ribeiro	1	F	25	Yes	No
104	Rnjak	1	M	53	No	No
105	Rodriguez J	1	M	84	Yes	Yes
106	Rodriguez-Pla	1	M	77	No	No
107	Rüfenacht	1	F	72	YES	Yes
108	Schenker C	1	M	61	No	No
109	Schreiber	1	F	67	No	No
110	Sepulcri	1	M	70	No	Yes
111	Shankar R	1	F	4	N/A	No
112	Spinicci M	1	F	26	No	No
113	Steiner	1	M	56	No	Yes

© 2023 Senefeld JW et al. *JAMA Network Open.*

No.	Study	patient no.	Sex	Age, y	Mechanical Ventilation?	Mortality?
		2	F	43	No	No
114	Szwebel	1	M	N/A	No	No
115	Taha	1	M	55	no	N/A
116	Trimarchi	1	M	24	No	No
117	Truong	1	M	21	N/A	No
118	Van Damme	1	M	37	Yes	No
119	van Oers	1	M	1	No	No
120	Wang	1	M	N/A	N/A	No
121	Wright	1	M	54	No	No
122	Yee	1	F	63	N/A	N/A
123	Zhang LB	1	F	50	No	No
124	Zhang LL	1	F	46	No	No
125	Zimmerli	1	M	74	No	No
126	Zimmermann	1	M	59	Yes	Yes

© 2023 Senefeld JW et al. *JAMA Network Open.*

eTable 7. Patient-Level Data (Part II)						
No.	Study	Discharge	WHO Disease Severity Score	CCP units	volume (ml) per unit	cumulative volume (ml)
1	Abid	No	5	N/A	N/A	N/A
		Yes	3	N/A	N/A	N/A
2	Adedoyin O	No	N/A	2	N/A	N/A
		Yes	N/A	1	N/A	N/A
3	Antony	N/A	N/A	1	200	200
4	Avanzato	N/A	N/A	2	200	400
5	Baang		N/A	N/A	N/A	N/A
6	Bakhsh A	Yes	N/A	4	N/A	N/A
7	Balashov	Yes	3	3	N/A	N/A
8	Basheer M	Yes	N/A	6	N/A	N/A
9	Bayrak	Yes	N/A	1	200	200
10	Belcari		N/A	6	600	3600
11	Bosnjak B	Yes	N/A	1	295	295
12	Bronstein	Yes	N/A	N/A	N/A	N/A
13	Bruiners N	Yes	N/A	3	500	1500
14	Buckland		3	2	N/A	N/A
15	Casarola G	Yes	3	3	N/A	N/A
16	Chen L	No	4	N/A	N/A	N/A
17	Choudhury A	Yes	N/A	N/A	N/A	N/A
18	Christensen J	No	N/A	N/A	N/A	N/A
19	Cinar O	Yes	N/A	2	200	400
20	Clark	Yes	N/A	N/A	N/A	800
21	Colombo	YES	N/A	2	300	600
22	Cusi MG	Yes	N/A	2	N/A	N/A
		Yes	N/A	2	N/A	N/A
		Yes	N/A	2	N/A	N/A
23	D'abramo	Yes	N/A	1	N/A	N/A
		Yes	N/A	1	N/A	N/A
		Yes	N/A	1	N/A	N/A
		Yes	N/A	1	N/A	N/A
		Yes	N/A	1	N/A	N/A
		Yes	N/A	1	N/A	N/A
		Yes	N/A	1	N/A	N/A
24	Dale M	Yes	5	2	N/A	N/A
		Yes	5	1	N/A	N/A
25	Delgado-Fernandez	Yes	N/A	1	600	600
		Yes	N/A	N/A	N/A	600
		Yes	N/A	N/A	N/A	300
26	Dell'Isola	N/A	2	N/A	N/A	N/A (10 mL/kg)
27	Deveci B	Yes	N/A	N/A	N/A	N/A

© 2023 Senefeld JW et al. *JAMA Network Open.*

No.	Study	Discharge	WHO Disease Severity Score	CCP units	volume (ml) per unit	cumulative volume (ml)
		Yes	N/A	N/A	N/A	N/A
		Yes	N/A	N/A	N/A	N/A
28	Di Palma	Yes	N/A	3	200	600
29	Erber J	Yes	5	5	200	1000
30	Ferrari	Yes	N/A	3	210	630
		Yes	N/A	3	210	630
		Yes	N/A	3	210	630
		Yes	N/A	3	210	630
		Yes	N/A	3	210	630
		Yes	N/A	3	210	630
		Yes	N/A	3	210	630
31	Franchini	N/A	N/A	3	300	900
		N/A	N/A	2	300	600
		N/A	N/A	2	300	600
		N/A	N/A	2	300	600
		N/A	N/A	2	300	600
		N/A	N/A	1	300	300
		N/A	N/A	2	300	600
		N/A	N/A	2	300	600
		N/A	N/A	2	300	600
32	Fung	Yes	N/A	1	200	200
		Yes	N/A	1	200	200
		Yes	N/A	1	200	200
		Yes	N/A	1	200	200
33	Furlan	Yes	4	N/A	N/A	900
		Yes	4	N/A	N/A	900
		Yes	3	N/A	N/A	900
		Yes	3	N/A	N/A	900
34	Gattuso G	Yes	2	N/A	N/A	N/A
35	Gordon O	Yes	N/A	N/A	N/A	5 mL/kg
		Yes	N/A	N/A	N/A	5 mL/kg
		Yes	N/A	N/A	N/A	5 mL/kg
		Yes	N/A	N/A	N/A	4 mL/kg
		Yes	N/A	N/A	N/A	5 mL/kg
		Yes	N/A	N/A	N/A	4 mL/kg
		Yes	N/A	N/A	N/A	5 mL/kg
		Yes	N/A	N/A	N/A	5 mL/kg
		Yes	N/A	N/A	N/A	5 mL/kg
36	Gupta	Yes	N/A	2	200	400
		Yes	N/A	2	200	400
		Yes	N/A	2	200	400
		Yes	N/A	2	200	400

© 2023 Senefeld JW et al. *JAMA Network Open.*

No.	Study	Discharge	WHO Disease Severity Score	CCP units	volume (ml) per unit	cumulative volume (ml)
		Yes	N/A	2	200	400
		Yes	N/A	2	200	400
		No	N/A	2	200	400
		Yes	N/A	2	200	400
		Yes	N/A	2	200	400
		Yes	N/A	2	200	400
37	Halfmann PJ	No	N/A	11	N/A	N/A
38	Hanssen JLJ	Yes	N/A	N/A	N/A	900
39	Hartman (Prophylaxis)	Yes	N/A	2	N/A	N
40	Hatzl		5	N/A	N/A	N/A
			5	N/A	N/A	N/A
41	Ho		5	N/A	N/A	N/A
			3	N/A	N/A	N/A
			2	N/A	N/A	N/A
			3	N/A	N/A	N/A
			N/A	N/A	N/A	N/A
42	Honjo	Yes	2	1	218	218
43	Hovey	Yes	2	1	199	199
44	Hughes CM	Yes	2	6	N/A	N/A
45	Iaboni	Yes	4	1	500	500
46	Jamir	Yes	4	3	200	600
47	Jassem J	Yes	N/A	N/A	N/A	400
48	Jiang J	Yes	N/A	N/A	N/A	400
49	Jin H	Yes	N/A	N/A	N/A	400
		Yes	N/A	N/A	N/A	400
		Yes	N/A	N/A	N/A	400
50	Karaolidou F	Yes	2	N/A	N/A	600
51	Karatas	Yes	N/A	N/A	N/A	N/A
52	Katz-Greenberg	Yes	N/A	N/A	N/A	N/A
		Yes	N/A	N/A	N/A	N/A
		Yes	N/A	N/A	N/A	N/A
		No	N/A	N/A	N/A	N/A
53	Keitel	Yes	2	6	250	1,500
54	Kemp	No	N/A	3	N/A	N/A
55	Kenig	Yes	N/A	2	N/A	N/A
		Yes	N/A	3	N/A	N/A
		Yes	N/A	2	N/A	N/A
		Yes	N/A	3	N/A	N/A
		Yes	N/A	2	N/A	N/A
56	Ketels	Yes	N/A	2	N/A	N/A
57	Khan AM	No	N/A	N/A	N/A	N/A

© 2023 Senefeld JW et al. *JAMA Network Open.*

No.	Study	Discharge	WHO Disease Severity Score	CCP units	volume (ml) per unit	cumulative volume (ml)
58	Khatamzas	N/A	N/A	5	N/A	N/A
59	Khatri	No	5	1	N/A	N/A
60	Kluger	N/A	N/A	3	N/A	N/A
		N/A	N/A	2	N/A	N/A
		Yes	N/A	2	N/A	N/A
61	Kremer AE	Yes	5	6	N/A	1800
		Yes	2	6	N/A	1800
		Yes	5	6	N/A	1800
62	Kutzler		N/A	N/A	N/A	N/A
			N/A	N/A	N/A	N/A
63	Lancman	Yes	N/A	2	N/A	N/A
64	Lang-Meli J	N/A	N/A	1	250	1-2 units (250 mL)
		N/A	N/A	2	250	1-2 units (250 mL)
		N/A	N/A	1	250	1-2 units (250 mL)
		N/A	N/A	2	250	1-2 units (250 mL)
		N/A	N/A	1	250	1-2 units (250 mL)
		N/A	N/A	2	250	1-2 units (250 mL)
		N/A	N/A	1	250	1-2 units (250 mL)
		N/A	N/A	2	250	1-2 units (250 mL)
		N/A	N/A	1	250	1-2 units (250 mL)
		N/A	N/A	2	250	1-2 units (250 mL)
		N/A	N/A	1	250	1-2 units (250 mL)
		N/A	N/A	2	250	1-2 units (250 mL)
		N/A	N/A	1	250	1-2 units (250 mL)
		N/A	N/A	2	250	1-2 units (250 mL)
		N/A	N/A	1	250	1-2 units (250 mL)
65	Lazzari L	Yes	4	4	200	800
66	Lemus HN	YES	N/A	2	200	400
67	Lima	no	N/A	N/A	N/A	N/A
		no	N/A	N/A	N/A	N/A
68	Lindemann 2021a (J Med Virol)	Yes	N/A	6	N/A	N/A
		Yes	N/A	3	N/A	N/A
69	Ljungquist O	Yes	6	N/A	N/A	N/A
		No	6	N/A	N/A	N/A
		Yes	4	N/A	N/A	N/A
		Yes	6	N/A	N/A	N/A
		Yes	4	N/A	N/A	N/A
		No	8	N/A	N/A	N/A
		No	10	N/A	N/A	N/A
		Yes	6	N/A	N/A	N/A
		Yes	5	N/A	N/A	N/A

© 2023 Senefeld JW et al. *JAMA Network Open.*

No.	Study	Discharge	WHO Disease Severity Score	CCP units	volume (ml) per unit	cumulative volume (ml)
		Yes	6	N/A	N/A	N/A
		Yes	6	N/A	N/A	N/A
		No	4	N/A	N/A	N/A
		No	8	N/A	N/A	N/A
70	London	Yes	N/A	1	400	400
71	Lubnow	Yes	N/A	3	N/A	N/A
72	Luetkens	Yes	N/A	1	N/A	200
73	Madariaga	Yes	N/A	N/A	N/A	~600
		Yes	N/A	1	300	~300
74	Malsy	Yes	N/A	6	N/A	N/A
75	Martens	Yes	4	N/A	N/A	N/A
76	Martínez-Barranco P	Yes	N/A	N/A	N/A	300
		no	N/A	2	300	600
		Yes	N/A	2	300	600
		yes	N/A	1	300	300
77	Martinez-Chincilla	Yes	N/A	1	N/A	N/A
		Yes	N/A	1	N/A	N/A
78	Martinot	Yes	3	N/A	N/A	800
79	McKemey		N/A	2	275	550
80	Mehta	No	N/A	N/A	N/A	N/A
		Yes	N/A	N/A	N/A	N/A
81	Mendes-Correa	Yes	4	N/A	N/A	N/A
82	Meyts		N/A	N/A	N/A	N/A
			N/A	N/A	N/A	N/A
			N/A	N/A	N/A	N/A
			N/A	N/A	N/A	N/A
			N/A	N/A	N/A	N/A
			N/A	N/A	N/A	N/A
83	Milosevic	Yes	3	4		0.5 mL/kg x 4
84	Mira	Yes	2	1	200	200
85	Mohseni	Yes	N/A	N/A	N/A	N/A
86	Monrad	N/A	N/A	N/A	N/A	1,800
87	Moore	Yes	2	1	200	200
88	Moutinho-Pereira	Yes	N/A	N/A	N/A	800
89	Naeem	Yes	N/A	1	N/A	N/A
		Yes	N/A	2	N/A	N/A
		Yes	N/A	1	N/A	N/A
90	Nguyen	Yes	N/A	1	N/A	N/A
91	Niu	Yes	N/A	2	200	400
92	Nussenblatt	No	N/A	N/A	N/A	N/A
93	Nyström	Yes	N/A	N/A	N/A	300

© 2023 Senefeld JW et al. *JAMA Network Open.*

No.	Study	Discharge	WHO Disease Severity Score	CCP units	volume (ml) per unit	cumulative volume (ml)
94	Oliva	Yes	N/A	3	300	900
		Yes	N/A	3	300	900
		Yes	N/A	3	300	900
		Yes	N/A	3	300	900
		No	N/A	1	300	300
		Yes	N/A	3	300	900
95	Ordaya EE	Yes	N/A	10	N/A	N/A
96	Ormazabal Velez	Yes	3	1	300	300
		Yes	4	2	300	600
97	Pal		4	1	200	200
			3	1	200	200
			4	2	200	400
			4	2	200	400
			5	1	200	200
			3	1	200	200
98	Pommeret F	Yes	N/A	2	N/A	N/A
		No	N/A	1	N/A	N/A
		Yes	N/A	2	N/A	N/A
		Yes	N/A	2	N/A	N/A
		Yes	N/A	2	N/A	N/A
99	Prasad	Yes	N/A	2	N/A	N/A
100	Rahman		3	N/A	N/A	N/A
			3	N/A	N/A	N/A
			4	N/A	N/A	N/A
			4	N/A	N/A	N/A
			4	N/A	N/A	N/A
			3	N/A	N/A	N/A
			4	N/A	N/A	N/A
			3	N/A	N/A	N/A
			3	N/A	N/A	N/A
			3	N/A	N/A	N/A
			3	N/A	N/A	N/A
			4	N/A	N/A	N/A
			3	N/A	N/A	N/A
101	Reuken		5	N/A	N/A	N/A
102	Rem	NO	N/A	N/A	N/A	N/A
103	Ribeiro	Yes	5	N/A	N/A	800
104	Rnjak	Yes	3	8	200	1,600
105	Rodriguez J	No	5	N/A	N/A	N/A
106	Rodriguez-Pla	Yes	3	2	N/A	N/A
107	Rüfenacht	NO	N/A	N/A	N/A	N/A
108	Schenker C	Yes	3	2	200	400

© 2023 Senefeld JW et al. *JAMA Network Open.*

No.	Study	Discharge	WHO Disease Severity Score	CCP units	volume (ml) per unit	cumulative volume (ml)
109	Schreiber	Yes	2	N/A	N/A	N/A
110	Sepulcri	No	N/A	N/A	N/A	N/A
111	Shankar R	Yes	N/A	N/A	N/A	15 mL / kg x 2 days
112	Spinicci M	No	N/A	2	200	400
113	Steiner	No	6	3	200	600
		Yes	5	2	200	400
114	Szwebel	Yes	2	4	N/A	N/A
115	Taha	N/A	N/A	2	N/A	N/A
116	Trimarchi	Yes	3	1	200	200
117	Truong		4	9	N/A	N/A
118	Van Damme	Yes	5	N/A	N/A	460
119	van Oers	Yes	2	N/A	N/A	20 mL/kg
120	Wang		N/A	N/A	N/A	N/A
121	Wright	Yes	3	N/A	N/A	200
122	Yee	N/A	N/A	N/A	N/A	200
123	Zhang LB	Yes	N/A	N/A	N/A	200
124	Zhang LL	N/A	2	N/A	N/A	200
125	Zimmerli	Yes	N/A	8	200	1600
126	Zimmermann	no	N/A	2	200	400

© 2023 Senefeld JW et al. *JAMA Network Open.*

eTable 7. Individual Patient Data (Part III)

No.	Study	Antibody Level	Antibody Test	Time from admission to transfusion (days)
1	Abid	N/A	N/A	10
		N/A	N/A	5
2	Adedoyin O	N/A	N/A	N/A
		N/A	N/A	N/A
3	Antony	N/A	N/A	N/A
4	Avanzato	1D: 2,560 (IgG), 2D: 5,120 (IgG)	ELISA	71
5	Baang	N/A	N/A	33
6	Bakhsh A	N/A	N/A	>4
7	Balashov	>1:160 (2), >1:80 (1)	N/A	N/A
8	Basheer M	>1:1000	ELISA	N/A
9	Bayrak	N/A	N/A	4
10	Belcari	640 nAb, others	VNT, serology	N/A
11	Bosnjak B	>1:100	pseudo nAb	N/A
12	Bronstein	N/A	N/A	12
13	Bruiners N	>1:160	nAb	N/A
14	Buckland	N/A	N/A	N/A
15	Casarola G	1:320, 1:160, 1:80	N/A	72
16	Chen L	N/A	N/A	1
17	Choudhury A	N/A	N/A	8
18	Christensen J	N/A	N/A	N/A
19	Cinar O		Euroimmun ELISA	1
20	Clark	N/A	N/A	43
21	Colombo	599	Ortho-VITROS	
22	Cusi MG	1:80	N/A	N/A
		1:80	N/A	N/A
		1:100	N/A	N/A
23	D'abramo	1/320		
		1/320		
		1/320		
		1/320		
		1/320		
		1/320		
		1/320		
24	Dale M	N/A	N/A	N/A
		N/A	N/A	N/A
25	Delgado-Fernandez	> 2.64	COVID-19 ELISA IgG	23 (1st admit), 4 (readmit)
		3.95	COVID-19 ELISA IgG	27 (1st admit), 14 (readmit)
		3.69	COVID-19 ELISA IgG	49 (1st admit), 2 (readmit)
26	Dell'Isola	1:20, 1:160	N/A	10-13
27	Deveci B	N/A	N/A	N/A

© 2023 Senefeld JW et al. *JAMA Network Open.*

No.	Study	Antibody Level	Antibody Test	Time from admission to transfusion (days)
		N/A	N/A	N/A
		N/A	N/A	N/A
28	Di Palma			49,50,51
29	Erber J	<10	IgG	20
30	Ferrari	N/A	N/A	N/A
		N/A	N/A	N/A
		N/A	N/A	N/A
		N/A	N/A	N/A
		N/A	N/A	N/A
		N/A	N/A	N/A
		N/A	N/A	N/A
31	Franchini	160-320	VNT	N/A
		320	VNT	N/A
		160	VNT	N/A
		640	VNT	N/A
		640	VNT	N/A
		160	VNT	N/A
		320	VNT	N/A
		640	VNT	N/A
		320-640	VNT	N/A
32	Fung	>1:400	ELISA	21
		>1:400	ELISA	2
		N/A	N/A	5
		>1:400	ELISA	2
33	Furlan	N/A	N/A	
		N/A	N/A	
		N/A	N/A	
		N/A	N/A	
34	Gattuso G	N/A	N/A	11
35	Gordon O	> 1: 320	Euroimmun ELISA	N/A
		> 1: 320	Euroimmun ELISA	N/A
		> 1: 320	Euroimmun ELISA	N/A
		> 1: 320	Euroimmun ELISA	N/A
		> 1: 320	Euroimmun ELISA	N/A
		> 1: 320	Euroimmun ELISA	N/A
		> 1: 320	Euroimmun ELISA	N/A
		> 1: 320	Euroimmun ELISA	N/A
		> 1: 320	Euroimmun ELISA	N/A
36	Gupta	>1:640	N/A	8
		>1:640	N/A	4
		>1:640	N/A	8
		>1:640	N/A	5
		>1:640	N/A	8

© 2023 Senefeld JW et al. *JAMA Network Open.*

No.	Study	Antibody Level	Antibody Test	Time from admission to transfusion (days)
		>1:640	N/A	6
		>1:640	N/A	5
		>1:640	N/A	4
		>1:640	N/A	4
		>1:640	N/A	3
37	Halfmann PJ			
38	Hanssen JLJ	> 60 alliance units/mL	ELISA	9
39	Hartman (Prophylaxis)	N/A	N/A	N/A
40	Hatzl	N/A	N/A	N/A
		N/A	N/A	N/A
41	Ho	N/A	N/A	N/A
		N/A	N/A	N/A
		N/A	N/A	N/A
		N/A	N/A	N/A
		N/A	N/A	N/A
42	Honjo	ID 50 > 5,000	ELISA	6
43	Hovey	N/A	N/A	11
44	Hughes CM	N/A	N/A	N/A
45	Iaboni	N/A	N/A	5
46	Jamir	> 1: 1000	N/A	N/A
47	Jassem J	N/A	N/A	88
48	Jiang J	>1:1000	ELISA	4
49	Jin H	> 1: 320	N/A	12
		> 1: 320	N/A	3
		> 1: 320	N/A	2
50	Karaolidou F	N/A	N/A	≥ 11
51	Karatas	> 1.1	Euroimmun ELISA	N/A
52	Katz-Greenberg	N/A	N/A	N/A
		N/A	N/A	N/A
		N/A	N/A	N/A
		N/A	N/A	N/A
53	Keitel	> 1:80	N/A	55, 57, 60, 62, 64
54	Kemp	N/A	Euroimmun	N/A
55	Kenig	N/A	Liasion Assay	N/A
		N/A	Liasion Assay	N/A
		N/A	Liasion Assay	N/A
		N/A	Liasion Assay	N/A
		Anti-SARS-CoV hyperimmune globulin	Liasion Assay	N/A
56	Ketels	N/A	N/A	14, 28
57	Khan AM	N/A	N/A	N/A
58	Khatamzas	N/A		N/A

© 2023 Senefeld JW et al. *JAMA Network Open.*

No.	Study	Antibody Level	Antibody Test	Time from admission to transfusion (days)
59	Khatri	N/A	N/A	N/A
60	Kluger	> 400 IU	Euroimmun	N/A
		N/A	N/A	N/A
		N/A	N/A	N/A
61	Kremer AE	61, 50 AU/mL	DiaSorin Clia	28
		81, 96 AU/mL	DiaSorin Clia	17
		51, 91 AU/mL	DiaSorin Clia	4
62	Kutzler	N/A	N/A	N/A
		N/A	N/A	N/A
63	Lancman	N/A	N/A	10
64	Lang-Meli J	>1:320 or >4	ELISA	N/A
		>1:320 or >5	ELISA	N/A
		>1:320 or >6	ELISA	N/A
		>1:320 or >7	ELISA	N/A
		>1:320 or >8	ELISA	N/A
		>1:320 or >9	ELISA	N/A
		>1:320 or >10	ELISA	N/A
		>1:320 or >11	ELISA	N/A
		>1:320 or >12	ELISA	N/A
		>1:320 or >13	ELISA	N/A
		>1:320 or >14	ELISA	N/A
		>1:320 or >15	ELISA	N/A
		>1:320 or >17	ELISA	N/A
		>1:320 or >18	ELISA	N/A
		>1:320 or >19	ELISA	N/A
65	Lazzari L	N/A	N/A	
66	Lemus HN	N/A		5, 13
67	Lima	N/A		N/A
		N/A		N/A
68	Lindemann 2021a (J Med Virol)	1/1280	ELISA; Euroimmun	N/A
		1/320	ELISA; Euroimmun	N/A
69	Ljungquist O	640	N/A	N/A
		412	N/A	N/A
		205	N/A	N/A
		503	N/A	N/A
		36	N/A	N/A
		253	N/A	N/A
		183	N/A	N/A
		297	N/A	N/A
		336	N/A	N/A
		290	N/A	N/A
		351	N/A	N/A

© 2023 Senefeld JW et al. *JAMA Network Open.*

No.	Study	Antibody Level	Antibody Test	Time from admission to transfusion (days)
		139	N/A	N/A
		95	N/A	N/A
70	London	1:160, 1:40	N/A	N/A
71	Lubnow	N/A	N/A	13
72	Luetkens	>1:1000	ELISA	1
73	Madariaga	1: 1,648	ELISA	8
		1: 2,921	ELISA	3
74	Malsy			4 readmit
75	Martens	N/A	N/A	N/A
76	Martínez-Barranco P	N/A		
		N/A		
		N/A		
		N/A		
77	Martinez-Chincilla	N/A	N/A	N/A
		N/A	N/A	
78	Martinot	>1.1	ELISA	62 - 65
79	McKemey	>100	VNT	N/A
80	Mehta	N/A	N/A	N/A
		N/A	N/A	N/A
81	Mendes-Correa	N/A	N/A	N/A
82	Meyts	N/A	N/A	N/A
		N/A	N/A	N/A
		N/A	N/A	N/A
		N/A	N/A	N/A
		N/A	N/A	N/A
		N/A	N/A	N/A
83	Milosevic	N/A	N/A	10 & 5 weeks
84	Mira	> 1:320	Vircell ELISA	23
85	Mohseni	N/A	N/A	N/A
86	Monrad	4.46, 8.24, 4.72, 5.44	Euroimmun ELISA	16
87	Moore	N/A	Ortho-VITROS	0
88	Moutinho-Pereira	N/A	N/A	102
89	Naeem	8.68	Abbott	7
		5.7, 8.15	Abbott	4
		5.67	Abbott	2
90	Nguyen	N/A	N/A	4
91	Niu	N/A	N/A	4
92	Nussenblatt	N/A	N/A	2
93	Nyström	N/A	N/A	35
94	Oliva			120
				65
				37

© 2023 Senefeld JW et al. *JAMA Network Open.*

No.	Study	Antibody Level	Antibody Test	Time from admission to transfusion (days)
				65
				13
				9
95	Ordaya EE	High	N/A	N/A
96	Ormazabal Velez			N/A
				N/A
97	Pal	N/A	ELISA	3
		N/A	ELISA	0
		N/A	ELISA	12
		800	ELISA	16
		3200	ELISA	3
		400	ELISA	1
98	Pommeret F			
99	Prasad	N/A	N/A	1
100	Rahman	N/A	N/A	N/A
		N/A	N/A	N/A
		N/A	N/A	N/A
		N/A	N/A	N/A
		N/A	N/A	N/A
		N/A	N/A	N/A
		N/A	N/A	N/A
		N/A	N/A	N/A
		N/A	N/A	N/A
		N/A	N/A	N/A
		N/A	N/A	N/A
		N/A	N/A	N/A
		N/A	N/A	N/A
101	Reuken	N/A	N/A	N/A
102	Rem			52
103	Ribeiro	1/1280 & 1/320	In house nAb	11, 11, 13, 16
104	Rnjak	between 108 to 890 IU/mL	N/A	48
105	Rodriguez J	N/A	N/A	11
106	Rodriguez-Pla	N/A	N/A	N/A
107	Rüfenacht			52
108	Schenker C	N/A	N/A	9
109	Schreiber	N/A	N/A	N/A
110	Sepulcri	N/A	ELISA	88
111	Shankar R	N/A	N/A	8-9

© 2023 Senefeld JW et al. *JAMA Network Open.*

No.	Study	Antibody Level	Antibody Test	Time from admission to transfusion (days)
112	Spinicci M	1/320	N/A	53
113	Steiner	1/320	N/A	
		1/320	N/A	
114	Szwebel	N/A	N/A	65
115	Taha			
116	Trimarchi	N/A	N/A	9
117	Truong	N/A	N/A	N/A
118	Van Damme	N/A	N/A	20
119	van Oers	N/A	N/A	6
120	Wang	N/A	N/A	N/A
121	Wright	N/A	N/A	3
122	Yee	N/A	N/A	29
123	Zhang LB	>10	N/A	10
124	Zhang LL	1:640	N/A	5
125	Zimmerli	High titer (4.43, 5.29, 7.67)	EUROIMMUN	72, 73, 84, 85, 94, 95, 111, 112
126	Zimmermann			69

© 2023 Senefeld JW et al. *JAMA Network Open.*

eTable 7. Individual Patient Data (Part IV)					
No.	Study	Time from symptoms to transfusion (days)	Rapid improvement (within 5 days)?	Follow-up (days)	ICU length of stay
1	Abid	33	N/A	N/A	N/A
		N/A	N/A	N/A	N/A
2	Adedoyin O	N/A	N/A	N/A	N/A
		N/A	N/A	N/A	N/A
3	Antony	N/A	N/A	N/A	N/A
4	Avanzato	N/A	No	N/A	N/A
5	Baang	40	N/A		
6	Bakhsh A	>12	N/A	N/A	
7	Balashov	47	No	N/A	N/A
8	Basheer M	N/A	No	N/A	N/A
9	Bayrak	9	No	N/A	N/A
10	Belcari	115	Yes	180	N/A
11	Bosnjak B	63	Yes	90	N/A
12	Bronstein	N/A	N/A	N/A	43
13	Bruiners N	39.54.61	N/A	N/A	N/A
14	Buckland	N/A	N/A		
15	Casarola G	N/A	Yes	N/A	N/A
16	Chen L	N/A	No	94	49
17	Choudhury A	12	Yes		14
18	Christensen J	N/A	No	11	N/A
19	Cinar O	5	N/A	N/A	N/A
20	Clark	50	Yes	69	N/A
21	Colombo	72	Yes	4	N/A
22	Cusi MG	N/A	N/A	N/A	N/A
		N/A	N/A	N/A	N/A
		N/A	N/A	N/A	N/A
23	D'abramo		N/A	N/A	92
			N/A	N/A	19
			N/A	N/A	19
			N/A	N/A	30
			N/A	N/A	15
			N/A	N/A	51
			N/A	N/A	6
24	Dale M	10	No	N/A	N/A
		5	No	N/A	N/A
25	Delgado-Fernandez	39	N/A	N/A	N/A
		36	No	N/A	N/A
		56	Yes	N/A	N/A
26	Dell'Isola	30-33	No	··	N/A
27	Deveci B	N/A	N/A	N/A	N/A

© 2023 Senefeld JW et al. *JAMA Network Open.*

No.	Study	Time from symptoms to transfusion (days)	Rapid improvement (within 5 days)?	Follow-up (days)	ICU length of stay
		N/A	N/A	N/A	N/A
		N/A	N/A	N/A	N/A
28	Di Palma	49	yes	150	N/A
29	Erber J	21	No	N/A	71
30	Ferrari	8	N/A	N/A	N/A
		7	N/A	N/A	N/A
		6	N/A	N/A	N/A
		9	N/A	N/A	N/A
		7	N/A	N/A	N/A
		10	N/A	N/A	N/A
		10	N/A	N/A	N/A
31	Franchini	10	Yes		
		11	Yes		
		18	No		
		19	No		
		9	Yes		
		11	Yes		
		1	Yes		
		3	Yes		
		15	Yes		
32	Fung	27	No	73	N/A
		4	Yes	15	N/A
		8	Yes	17	N/A
		4	No	61	N/A
33	Furlan		No		N/A
			No		N/A
			No		N/A
			No	N/A	N/A
34	Gattuso G	11	N/A	107	N/A
35	Gordon O	5	N/A	N/A	N/A
		5	N/A	N/A	N/A
		4	N/A	N/A	N/A
		5	N/A	N/A	N/A
		5	N/A	N/A	N/A
		3	N/A	N/A	N/A
		7	N/A	N/A	N/A
		2	N/A	N/A	N/A
		5	N/A	N/A	N/A
36	Gupta	11	N/A	7	N/A
		6	N/A	7	N/A
		10	N/A	7	N/A
		9	N/A	7	N/A

© 2023 Senefeld JW et al. *JAMA Network Open.*

No.	Study	Time from symptoms to transfusion (days)	Rapid improvement (within 5 days)?	Follow-up (days)	ICU length of stay
		11	N/A	7	N/A
		8	N/A	7	N/A
		10	N/A	7	N/A
		7	N/A	7	N/A
		6	N/A	7	N/A
		7	N/A	7	N/A
37	Halfmann PJ	4 - 6 - 14 - 16 months	NO		N/A
38	Hanssen JLJ	29	No	54	N/A
39	Hartman (Prophylaxis)	N/A	No	61	N/A
40	Hatzl	N/A	N/A		
		N/A	N/A		
41	Ho	N/A	N/A		
		N/A	N/A		
		N/A	N/A		
		N/A	N/A		
		N/A	N/A		
42	Honjo	33	Yes	4	N/A
43	Hovey	18	Yes	14	N/A
44	Hughes CM	8	N/A	N/A	N/A
45	Iaboni	12	Yes	N/A	8
46	Jamir	4	No	N/A	24
47	Jassem J	88	Yes	62	N/A
48	Jiang J	26	N/A	14	N/A
49	Jin H	22	Yes	15	N/A
		16	Yes	4	N/A
		44	Yes	22	N/A
50	Karaolidou F	≥ 13	Yes	90	N/A
51	Karatas	40	No	N/A	N/A
52	Katz-Greenberg	N/A	N/A	N/A	N/A
		N/A	N/A	N/A	N/A
		N/A	N/A	N/A	N/A
		N/A	N/A	N/A	N/A
53	Keitel	55, 57, 60, 62, 64	No	138	N/A
54	Kemp	63	No	N/A	N/A
55	Kenig	40	N/A	N/A	N/A
		45	Yes	N/A	N/A
		60	Yes	N/A	N/A
		35	No	N/A	N/A
		7	N/A	N/A	N/A
56	Ketels	N/A	No	N/A	77

© 2023 Senefeld JW et al. *JAMA Network Open.*

No.	Study	Time from symptoms to transfusion (days)	Rapid improvement (within 5 days)?	Follow-up (days)	ICU length of stay
57	Khan AM	N/A	No	N/A	N/A
58	Khatamzas	40;65,95,110,120	N/A	N/A	N/A
59	Khatri	N/A	N/A	N/A	N/A
60	Kluger	N/A	N/A	N/A	N/A
		N/A	No	N/A	N/A
		N/A	Yes	N/A	N/A
61	Kremer AE	28	No	N/A	N/A
		27	No	N/A	N/A
		5	Yes	N/A	N/A
62	Kutzler	N/A	Yes		
		N/A	N/A		
63	Lancman	80	Yes	N/A	N/A
64	Lang-Meli J	13	N/A	N/A	N/A
		48	N/A	N/A	N/A
		13	N/A	N/A	N/A
		8	N/A	N/A	N/A
		132	N/A	N/A	N/A
		61	N/A	N/A	N/A
		34	N/A	N/A	N/A
		51	N/A	N/A	N/A
		27	N/A	N/A	N/A
		17	N/A	N/A	N/A
		6	N/A	N/A	N/A
		23	N/A	N/A	N/A
		10	N/A	N/A	N/A
		10	N/A	N/A	N/A
		5	N/A	N/A	N/A
65	Lazzari L		No	N/A	75
66	Lemus HN	5	Yes	N/A	
67	Lima	N/A	N/A	N/A	17
		N/A	N/A	N/A	21
68	Lindemann 2021a (J Med Virol)	3	Yes	28	
		13	Yes	16	
69	Ljungquist O	60	N/A	N/A	N/A
		20	N/A	N/A	N/A
		29	N/A	N/A	N/A
		21	N/A	N/A	N/A
		6	N/A	N/A	N/A
		25	N/A	N/A	N/A
		6	N/A	N/A	N/A
		25	N/A	N/A	N/A

© 2023 Senefeld JW et al. *JAMA Network Open.*

No.	Study	Time from symptoms to transfusion (days)	Rapid improvement (within 5 days)?	Follow-up (days)	ICU length of stay
		13	N/A	N/A	N/A
		21	N/A	N/A	N/A
		30	N/A	N/A	N/A
		40	N/A	N/A	N/A
		8	N/A	N/A	N/A
70	London	71	Yes	N/A	N/A
71	Lubnow	15	Yes	54	N/A
72	Luetkens	2	Yes	19	N/A
73	Madariaga	9	Yes	N/A	N/A
		13	Yes	N/A	N/A
74	Malsy	approx. 90	NO	20	N/A
75	Martens	N/A	N/A	N/A	N/A
76	Martínez-Barranco P	88	yes	15	N/A
		28, 48	no		N/A
		21.32	yes	17	N/A
		30	yes	10	N/A
77	Martinez-Chincilla	91	N/A	49	N/A
		42	Yes	16	N/A
78	Martinot	66 - 69	1, 100%	N/A	N/A
79	McKemey	100	N/A	155	N/A
80	Mehta	N/A	N/A	N/A	33
		N/A	N/A	N/A	41
81	Mendes-Correa	N/A	N/A	N/A	N/A
82	Meyts	N/A	N/A		
		N/A	N/A		
		N/A	N/A		
		N/A	N/A		
		N/A	N/A		
		N/A	N/A		
83	Milosevic	15 & 6 weeks	No	56	N/A
84	Mira	37	Yes	N/A	N/A
85	Mohseni	N/A	N/A	N/A	N/A
86	Monrad	58	No	263	N/A
87	Moore	88	Yes	7	N/A
88	Moutinho-Pereira	121	Yes	56	N/A
89	Naeem	9	Yes	N/A	N/A
		4	No	N/A	25
		7	Yes	N/A	16
90	Nguyen	N/A	N/A	26	
91	Niu	N/A	Yes	15	
92	Nussenblatt	13	N/A	N/A	N/A

© 2023 Senefeld JW et al. *JAMA Network Open.*

No.	Study	Time from symptoms to transfusion (days)	Rapid improvement (within 5 days)?	Follow-up (days)	ICU length of stay
93	Nyström	N/A		42	
94	Oliva	120, 122,124	Yes	43	170
		65, 67, 69	Yes	33	93
		37, 39, 41			
		65, 67, 69			
		13			
		9, 11, 13			
95	Ordaya EE	N/A	No	N/A	N/A
96	Ormazabal Velez	85		120	
		78		112	
97	Pal	4	Yes		
		5	Yes		
		13	Yes		
		17	Yes		
		18	No		
		5	No		
98	Pommeret F				
99	Prasad	7	Yes	20	20
100	Rahman	7	N/A		
		5	N/A		
		6	N/A		
		15	N/A		
		9	N/A		
		11	N/A		
		31	N/A		
		12	N/A		
		7	N/A		
		11	N/A		
		4	N/A		
		7	N/A		
		8	N/A		
101	Reuken	N/A	Yes		
102	Rem	52	NO		
103	Ribeiro	11, 11, 13, 16	Yes	N/A	N/A
104	Rnjak	48	No	N/A	N/A
105	Rodriguez J	18	No	N/A	N/A
106	Rodriguez-Pla	N/A	N/A	N/A	N/A

© 2023 Senefeld JW et al. *JAMA Network Open.*

No.	Study	Time from symptoms to transfusion (days)	Rapid improvement (within 5 days)?	Follow-up (days)	ICU length of stay
107	Rüfenacht	52	NO	N/A	N/A
108	Schenker C	31	No	N/A	N/A
109	Schreiber	N/A	N/A	N/A	N/A
110	Sepulcri	N/A	No	N/A	271
111	Shankar R	N/A	N/A	N/A	19
112	Spinicci M	63	no	N/A	
113	Steiner				
					127
114	Szwebel	N/A	Yes	N/A	N/A
115	Taha	54, 55	yes	N/A	N/A
116	Trimarchi	N/A	N/A	16	N/A
117	Truong	N/A	N/A		
118	Van Damme	27	Yes	33	N/A
119	van Oers	..	..	..	..
120	Wang	N/A	N/A		
121	Wright	25	Yes	9	9
122	Yee	38	Yes	N/A	36
123	Zhang LB	17	Yes	7	N/A
124	Zhang LL	5	1, 100%	14	N/A
125	Zimmerli	N/A	No	43	N/A
126	Zimmermann	69	no		122

© 2023 Senefeld JW et al. *JAMA Network Open.*

**eTable 7**. Individual Patient Data (Part V)					
No.	Study	IVIG	Steroids	remdesivir	(hydroxy)chloroquine
1	Abid	No	No	Yes	No
		No	Yes	No	No
2	Adedoyin O		Yes		
3	Antony	No		No	Yes
4	Avanzato	Yes	No	No	No
5	Baang	No	Yes	Yes	NO
6	Bakhsh A		Yes		
7	Balashov		Yes		
8	Basheer M	Yes		Yes	
9	Bayrak	No	Yes	No	No
10	Belcari	No	Yes	No	No
11	Bosnjak B	Yes	No	No	No
12	Bronstein	Yes		Yes	
13	Bruiners N	No	Yes	Yes	Yes
14	Buckland	NO	NO	Yes	Yes
15	Casarola G	Yes	Yes	Yes	
16	Chen L	No	Yes	No	No
17	Choudhury A	No	Yes	Yes	Yes
18	Christensen J	No	No	No	Yes
19	Cinar O	No	No	No	No
20	Clark	No	Yes	No	No
21	Colombo	Yes	YES	Yes	NO
22	Cusi MG	No	No	No	No
		No	No	No	No
		No	No	No	No
23	D'abramo		Yes	Yes	NO
			Yes	Yes	NO
			Yes	Yes	NO
			Yes	Yes	NO
			Yes	Yes	
			Yes	Yes	
			Yes	Yes	
24	Dale M		Yes		Yes
			Yes		Yes
25	Delgado-Fernandez	Yes	Yes	Yes	Yes
		No	Yes	No	No
		Yes	No	No	No
26	Dell'Isola	No	Yes	Yes	No
27	Deveci B	Yes			

© 2023 Senefeld JW et al. *JAMA Network Open.*

No.	Study	IVIG	Steroids	remdesivir	(hydroxy)chloroquine
		Yes			
28	Di Palma		Yes	Yes	no
29	Erber J			Yes	
30	Ferrari		Yes		Yes
			Yes		Yes
			Yes		Yes
			Yes		Yes
			Yes		Yes
					Yes
			Yes		Yes
31	Franchini	No	Yes	Yes	no
		No	Yes	No	no
		No	Yes	No	no
		No	Yes	No	no
		No	Yes	Yes	no
		No	Yes	NO	no
		No	Yes	No	no
		No	Yes	No	no
		Yes	Yes	No	no
32	Fung	No	Yes	No	Yes
		No	No	No	No
		No	No	Yes	No
		No	No	No	No
33	Furlan		Yes	Yes	
			Yes	Yes	
			Yes	Yes	
			Yes	Yes	
34	Gattuso G		Yes		
35	Gordon O	No	No	No	No
		Yes	No	No	No
		No	No	No	No
		No	Yes	Yes	No
		No	No	No	No
		Yes	No	No	No
		Yes	No	No	No
		No	No	No	No
		No	No	No	No
36	Gupta		Yes	Yes	
			Yes	Yes	
			Yes	Yes	
			Yes	Yes	
			Yes	Yes	
			Yes	Yes	

© 2023 Senefeld JW et al. *JAMA Network Open.*

No.	Study	IVIG	Steroids	remdesivir	(hydroxy)chloroquine
			Yes	Yes	
			Yes	Yes	
			Yes	Yes	
			Yes	Yes	
37	Halfmann PJ	Yes	Yes	Yes	no
38	Hanssen JLJ	Yes	Yes	Yes	
39	Hartman (Prophylaxis)				
40	Hatzl	No	Yes	No	Yes
		No	Yes	No	Yes
41	Ho	No	No	No	Yes
		No	Yes	No	Yes
		No	No	No	No
		No	No	No	No
		No	No	No	No
42	Honjo	Yes			Yes
43	Hovey	Yes	N/A	N/A	N/A
44	Hughes CM		Yes		
45	Iaboni	Yes	Yes	Yes	
46	Jamir		Yes	Yes	
47	Jassem J	Yes		Yes	
48	Jiang J		Yes		
49	Jin H	Yes		Yes	
		Yes			
		Yes			
50	Karaolidou F				
51	Karatas				Yes
52	Katz-Greenberg				
53	Keitel			Yes	
54	Kemp	No		Yes	
55	Kenig			Yes	
				Yes	
				Yes	
				Yes	
		Yes		Yes	
56	Ketels		yes	no	no
57	Khan AM	No		Yes	
58	Khatamzas				
59	Khatri				
60	Kluger	N/A	N/A	N/A	N/A

© 2023 Senefeld JW et al. *JAMA Network Open.*

No.	Study	IVIG	Steroids	remdesivir	(hydroxy)chloroquine
		N/A	N/A	N/A	N/A
		N/A	N/A	N/A	N/A
61	Kremer AE	No	No	No	Yes
		Yes	No	No	Yes
		No	No	No	No
62	Kutzler	No	No	No	No
		No	Yes	No	No
63	Lancman	Yes	No	Yes	Yes
64	Lang-Meli J				
				Yes	
			Yes	Yes	
			Yes		
				Yes	
				Yes	
			Yes	Yes	
			Yes	Yes	
			Yes	Yes	
65	Lazzari L		Yes	Yes	
66	Lemus HN	Yes	Yes	NO	NO
67	Lima		yes	no	no
			Yes	no	no
68	Lindemann 2021a (J Med Virol)		no	no	no
			no	no	no
69	Ljungquist O		Yes	No	
			Yes	No	
			Yes	No	
			Yes	Yes	
			No	No	
			Yes	Yes	
			Yes	Yes	
			Yes	Yes	
			Yes	Yes	
			Yes	Yes	
			Yes	Yes	
			Yes	Yes	
			Yes	Yes	

© 2023 Senefeld JW et al. *JAMA Network Open.*

No.	Study	IVIG	Steroids	remdesivir	(hydroxy)chloroquine
70	London		Yes		Yes
71	Lubnow		Yes		
72	Luetkens				
73	Madariaga				
74	Malsy	NO	NO	Yes	NO
75	Martens		Yes	Yes	
76	Martínez-Barranco P	yes	yes	yes	Yes
		no	Yes	Yes	
		yes	Yes	no	no
		no	Yes	No	no
77	Martinez-Chincilla	No	Yes	Yes	No
		No	Yes	Yes	No
78	Martinot	Yes	Yes	Yes	Yes
79	McKemey	No	Yes	Yes	NO
80	Mehta		Yes	Yes	
			Yes	Yes	
81	Mendes-Correa	Yes	Yes		
82	Meyts	yes	Yes	No	Yes
		yes	Yes	No	Yes
		NO	Yes	Yes	NO
		yes	No	Yes	NO
		yes	No	No	Yes
		NO	No	No	Yes
83	Milosevic		Yes		Yes
84	Mira	Yes			Yes
85	Mohseni			Yes	
86	Monrad			Yes	
87	Moore				
88	Moutinho-Pereira		Yes		
89	Naeem		Yes		
			Yes	Yes	
			Yes	Yes	
90	Nguyen				
91	Niu	Yes			
92	Nussenblatt	Yes	Yes	Yes	
93	Nyström	Yes	Yes	no	
94	Oliva		Yes	Yes	No
			Yes	Yes	No
			Yes	Yes	No
			Yes	Yes	No
			Yes	Yes	No

© 2023 Senefeld JW et al. *JAMA Network Open.*

No.	Study	IVIG	Steroids	remdesivir	(hydroxy)chloroquine
			Yes	Yes	No
95	Ordaya EE		Yes	Yes	
96	Ormazabal Velez		yes	no	Yes
			yes	yes	yes
97	Pal	No	No	Yes	No
		No	No	Yes	No
		No	No	No	No
		No	No	No	No
		No	No	Yes	No
		No	No	No	No
98	Pommeret F				
99	Prasad	No	No	No	No
100	Rahman	N/A	N/A	N/A	N/A
		N/A	N/A	N/A	N/A
		N/A	N/A	N/A	N/A
		N/A	N/A	N/A	N/A
		N/A	N/A	N/A	N/A
		N/A	N/A	N/A	N/A
		N/A	N/A	N/A	N/A
		N/A	N/A	N/A	N/A
		N/A	N/A	N/A	N/A
		N/A	N/A	N/A	N/A
		N/A	N/A	N/A	N/A
		N/A	N/A	N/A	N/A
		N/A	N/A	N/A	N/A
101	Reuken	NO	NO	Yes	NO
102	Rem	NO	Yes	Yes	NO
103	Ribeiro		Yes		
104	Rnjak		Yes		
105	Rodriguez J		Yes	Yes	Yes
106	Rodriguez-Pla		Yes	Yes	
107	Rüfenacht	NO	Yes	Yes	NO
108	Schenker C		Yes	Yes	
109	Schreiber		Yes	Yes	
110	Sepulcri	Yes	Yes	Yes	Yes
111	Shankar R	Yes	Yes	Yes	
112	Spinicci M		yes		
113	Steiner	Yes	Yes	No	No

© 2023 Senefeld JW et al. *JAMA Network Open.*

No.	Study	IVIG	Steroids	remdesivir	(hydroxy)chloroquine
		Yes	Yes	No	No
114	Szwebel		Yes		
115	Taha	no	Yes	Yes	
116	Trimarchi		Yes		
117	Truong	NO	NO	Yes	NO
118	Van Damme		Yes		
119	van Oers	Yes			
120	Wang	N/A	N/A	N/A	N/A
121	Wright				Yes
122	Yee				
123	Zhang LB		Yes		Yes
124	Zhang LL				
125	Zimmerli				
126	Zimmermann		Yes	Yes	no

© 2023 Senefeld JW et al. *JAMA Network Open.*

eTable 7. Individual Patient Data (Part VI)			
No.	Study	Antibiotics	Concomitant Therapies
1	Abid	Yes	Remdesivir
2	Adedoyin O		Tacrolimus and mycophenolate, bamlanivimab and etesevimab
			Ocrelizumab
3	Antony	Yes	cefepime, hydrochloroquine,
4	Avanzato	No	IVIG
5	Baang	N/A	rituximab, Chemotherapy
6	Bakhsh A	Yes	
7	Balashov	Yes	
8	Basheer M	Yes	
9	Bayrak	Yes	Meropenem, methylprednisolone,
10	Belcari	Yes	rituximab + benetoclax
11	Bosnjak B	N/A	G-CSF, CAR-T
12	Bronstein	Yes	Fludarabine, cyclophosphamide, rituximab
13	Bruiners N	Yes	Rituximab, methotrexate
14	Buckland	Yes	
15	Casarola G		
16	Chen L	Yes	meropenem, corticosteroids
17	Choudhury A	Yes	hydroxychloroquine, piperacillin-tazobactam, teicolplanin, dexamethasone, remdesivir
18	Christensen J	No	hydroxychloroquine
19	Cinar O	Yes	meropenem
20	Clark	No	prednisone,
21	Colombo	Yes	R-CHOP
22	Cusi MG	No	N/A
		No	N/A
		No	N/A
23	D'abramo		RITUXIMAB
			obinutuzumab
			RITUXIMAB
			RITUXIMAB
			RITUXIMAB
			RITUXIMAB
			RITUXIMAB
24	Dale M	Yes	
		Yes	
25	Delgado-Fernandez	Yes	hydroxychloroquine/azithromycin (H/A), cefditoren, ceftriaxone, tocilizumab, meropenem, remdesivir, dexamethasone
		Yes	tocilizumab, dexamethasone, cefepime, voriconazole, meropenem
		Yes	cefepime,
26	Dell'Isola	Yes	Cefpodoxime, remdesivir, prednisone,
27	Deveci B	Yes	Favipiravir

© 2023 Senefeld JW et al. *JAMA Network Open.*

No.	Study	Antibiotics	Concomitant Therapies
		Yes	Favipiravir
		Yes	
28	Di Palma	Yes	inotuzumab
29	Erber J		
30	Ferrari		Yes
			Yes
			Yes
			Yes
			Yes
			Yes
			Yes
31	Franchini	Yes	rituximab
		yes	
		Yes	
		yes	
		Yes	
		yes	metotrexate
		Yes	rituximab
		yes	rituximab
		Yes	anankira, ruxolitinib
32	Fung	Yes	tocilizumab, lopinavir/ritonavir, methylprednisolone, antibiotics, hydroxychloroquine
		No	N/A
		No	remdesivir
		No	N/A
33	Furlan		
34	Gattuso G		
35	Gordon O	No	N/A
		No	IVIG
		No	Infliximab
		No	dexamethasone, remdesivir
		No	N/A
		No	IVIG
		No	IVIG
		No	N/A
		No	N/A
36	Gupta		methylprednisolone, remdesivir
			methylprednisolone, remdesivir
			methylprednisolone, remdesivir
			methylprednisolone, remdesivir
			methylprednisolone, remdesivir

© 2023 Senefeld JW et al. *JAMA Network Open.*

No.	Study	Antibiotics	Concomitant Therapies
			methylprednisolone, remdesivir
			methylprednisolone, remdesivir
			methylprednisolone, remdesivir
			methylprednisolone, remdesivir
			methylprednisolone, remdesivir
37	Halfmann PJ	Yes	N/A
38	Hanssen JLJ	Yes	voriconazole, ibrutinib
39	Hartman (Prophylaxis)	Yes	
40	Hatzl	Yes	tocilizumab
		Yes	tocilizumab
41	Ho	Yes	
		Yes	
		Yes	
		Yes	
		No	
42	Honjo		
43	Hovey	Yes	
44	Hughes CM	Yes	
45	Iaboni		
46	Jamir	Yes	
47	Jassem J	Yes	obinutuzumab
48	Jiang J	Yes	
49	Jin H	Yes	
		Yes	
		Yes	
50	Karaolidou F	Yes	enoxaparin
51	Karatas		
52	Katz-Greenberg		
53	Keitel	Yes	
54	Kemp	No	remdesivir
55	Kenig		remdesivir, antibiotics and/or steroid
		Yes	remdesivir, ertapenem, azithromycin
			remdesivir, antibiotics and/or steroid
			remdesivir, antibiotics and/or steroid
			remdesivir, antibiotics and/or steroid
56	Ketels	yes	emtricitabine/tenofovir and dolutegravir
57	Khan AM	Yes	chemotherapy (liposomal daunorubicin 44 mg/m2 and cytarabine 100 mg/m2 on days 1, 3, and 5), cefepime, vancomycin, remdesivir, metronidazole
58	Khatamzas		

© 2023 Senefeld JW et al. *JAMA Network Open.*

No.	Study	Antibiotics	Concomitant Therapies
59	Khatri	Yes	
60	Kluger	N/A	N/A
		N/A	N/A
		N/A	N/A
61	Kremer AE	No	Hydroxychloroquine
		Yes	oseltamivir, IVIG, hydroxychloroquine, antibiotics
		Yes	antibiotics
62	Kutzler	Yes	
		Yes	tocilizumab
63	Lancman	Yes	Rituximab, cytarabine, dasatinib
64	Lang-Meli J		
		Yes	
		Yes	
		Yes	
		Yes	
		Yes	
		Yes	
		Yes	
		Yes	
		Yes	
65	Lazzari L	Yes	
66	Lemus HN	NO	TAMOXIFENE
67	Lima	no	Tacrolimus, prednisone
		no	Tacrolimus, prednisone, tocilizumab
68	Lindemann 2021a (J Med Virol)	no	
		no	
69	Ljungquist O		Enoxaparine
			Enoxaparine
			Tinazaparin
			Enoxaparine
			Enoxaparine
			Enoxaparine
			Enoxaparine
			Dalteparin
			Enoxaparine
			Enoxaparine
			Enoxaparine

© 2023 Senefeld JW et al. *JAMA Network Open.*

No.	Study	Antibiotics	Concomitant Therapies
			Enoxaparine
			Enoxaparine
70	London		
71	Lubnow	Yes	Anakinra
72	Luetkens		
73	Madariaga		tocilizumab, anakinra
74	Malsy	Yes	obinutuzumab
75	Martens	Yes	
76	Martínez-Barranco P	Yes	
		Yes	
		yes	Mesalazine and rifaximin
		Yes	rituximab, full-dose anticoagulation
77	Martinez-Chincilla	Yes	Tocilizumab, rituximab
		Yes	Rituximab
78	Martinot	Yes	
79	McKemey	Yes	rituximab
80	Mehta	Yes	
		Yes	
81	Mendes-Correa	Yes	
82	Meyts	Yes	
		Yes	
		Yes	tocilizumab
		Yes	
		Yes	
		Yes	
83	Milosevic	Yes	
84	Mira	Yes	
85	Mohseni		
86	Monrad	Yes	
87	Moore		Anti-CD20
88	Moutinho-Pereira	Yes	
89	Naeem	Yes	
		Yes	Azathioprine, tacrolimus
		Yes	
90	Nguyen		
91	Niu	Yes	
92	Nussenblatt	Yes	CART
93	Nyström	yes	
94	Oliva	Yes	rituximab

© 2023 Senefeld JW et al. *JAMA Network Open.*

No.	Study	Antibiotics	Concomitant Therapies
95	Ordaya EE	Yes	
96	Ormazabal Velez		
		Yes	Rituximab
97	Pal	No	
		No	
		No	
		Yes	
		No	
		No	
98	Pommeret F		
99	Prasad	Yes	Enoxaparine, rituximab
100	Rahman	N/A	
		N/A	
		N/A	
		N/A	
		N/A	
		N/A	
		N/A	
		N/A	
		N/A	
		N/A	
		N/A	
		N/A	
		N/A	
101	Reuken	No	infliximab
102	Rem	Yes	RITUXIMAB
103	Ribeiro	Yes	
104	Rnjak	Yes	
105	Rodriguez J	Yes	
106	Rodriguez-Pla	Yes	
107	Rüfenacht	Yes	RITUXIMAB
108	Schenker C	Yes	
109	Schreiber	Yes	
110	Sepulcri	Yes	rituximab, bendamustine, cytarabine
111	Shankar R	Yes	Voriconazole (antifungal)
112	Spinicci M	yes	Enoxaparine

© 2023 Senefeld JW et al. *JAMA Network Open.*

No.	Study	Antibiotics	Concomitant Therapies
113	Steiner		
114	Szwebel	Yes	
115	Taha	Yes	(R-CHOP)
116	Trimarchi	Yes	
117	Truong	No	CAR-T
118	Van Damme	Yes	
119	van Oers	Yes	
120	Wang	N/A	
121	Wright	Yes	
122	Yee		
123	Zhang LB		ciclosporin
124	Zhang LL		antiviral, arbidol, oseltamivir, ribavirin, interferon-alpha-2b
125	Zimmerli		
126	Zimmermann	yes	

© 2023 Senefeld JW et al. *JAMA Network Open.*

eTable 7. Individual Patient Data (Part VII)				
No.	Study	Primary or Secondary	Category	Condition
1	Abid	Secondary	Haematological Malignancy	MDS
		Secondary	Haematological Malignancy	MM
2	Adedoyin O	Secondary	Solid Organ Transplant	KTX
		Secondary	Autoimmune disorder	MS
3	Antony	Secondary	Solid Organ Transplant	KTX, OLT
4	Avanzato	Secondary	Haematological Malignancy	CLL
5	Baang	Secondary	Hematologic malignancy	LNH
6	Bakhsh A	Secondary	Solid Organ Transplant	HTX
7	Balashov	Secondary	Haematological Malignancy	HSCT
8	Basheer M	Secondary	Haematological Malignancy	NHL
9	Bayrak	Secondary	Solid Organ Transplant	KTX
10	Belcari	Secondary	Hematologic malignancy	CLL
11	Bosnjak B	Secondary	Haematological Malignancy	NHL
12	Bronstein	Secondary	Haematological Malignancy	CLL
13	Bruiners N	Secondary	Autoimmune disorder	Multiple autoimmune syndrome
14	Buckland	Primary	humoral	agammaglobulinemia
15	Casarola G	Secondary	Haematological Malignancy	NHL
16	Chen L	Secondary	Solid Organ Transplant	KTX
17	Choudhury A	Secondary	Solid Organ Transplant	OLT
18	Christensen J	Secondary	Solid Organ Transplant	KTX
19	Cinar O	Secondary	Haematological Malignancy	MDS
20	Clark	Secondary	Haematological Malignancy	NHL
21	Colombo	Secondary	Haematological Malignancy	NHL
22	Cusi MG	Secondary	Solid Organ Transplant	LTX
		Secondary	Haematological Malignancy	NHL

© 2023 Senefeld JW et al. *JAMA Network Open.*

No.	Study	Primary or Secondary	Category	Condition
		Secondary	Haematological Malignancy	CLL
23	D'abramo	Secondary	Haematological Malignancy	NHL
		Secondary	Haematological Malignancy	NHL
		Secondary	Haematological Malignancy	NHL
		Secondary	Haematological Malignancy	NHL
		Secondary	Haematological Malignancy	NHL
		Secondary	Haematological Malignancy	NHL
		Secondary	Haematological Malignancy	NHL
24	Dale M	Secondary	Solid Organ Transplant	LTX
		Secondary	Solid Organ Transplant	LTX
25	Delgado-Fernandez	Primary	humoral	XLA
		Secondary	Haematological Malignancy	NHL
		Secondary	Haematological Malignancy	NHL
26	Dell'Isola	Secondary	Haematological Malignancy	ALL
27	Deveci B	Secondary	Haematological Malignancy	NHL
		Secondary	Haematological Malignancy	CLL
		Secondary	Haematological Malignancy	NHL
28	Di Palma	Secondary	Haematological Malignancy	ALL
29	Erber J	Secondary	Haematological Malignancy	NHL
30	Ferrari	Secondary	Haematological Malignancy	NHL
		Secondary	Haematological Malignancy	NHL
		Secondary	Haematological Malignancy	NHL
		Secondary	Haematological Malignancy	IMF
		Secondary	Haematological Malignancy	NHL
		Secondary	Haematological Malignancy	AML
		Secondary	Haematological Malignancy	CLL

© 2023 Senefeld JW et al. *JAMA Network Open.*

No.	Study	Primary or Secondary	Category	Condition
31	Franchini	Secondary	Solid cancer	thymoma
		Secondary	Hematologic malignancy	NHL
		Secondary	Solid cancer	prostate cancer
		Secondary	solid cancer	lung cancer
		Secondary	Hematologic malignancy	CLL
		Secondary	Autoimmune Disorder	rheumatoid arthritis
		Secondary	Hematologic malignancy	NHL
		Secondary	Hematologic malignancy	CLL
		Secondary	Hematologic malignancy	PHLH
32	Fung	Secondary	Solid Organ Transplant	RTX
		Secondary	Solid Organ Transplant	RTX
		Secondary	Solid Organ Transplant	LTX
		Secondary	Haematological Malignancy	CML
33	Furlan	Secondary	Haematological Malignancy	NHL
		Secondary	Haematological Malignancy	NHL
		Secondary	Haematological Malignancy	NHL
		Secondary	Haematological Malignancy	NHL
34	Gattuso G	Secondary	Haematological Malignancy	NHL
35	Gordon O	Secondary	Solid cancer	DICER1-associated parietal Sarcoma
		Primary	Immunodeficiency	Immunodeficiency CARD11 mutation
		Secondary	Autoimmune disorder	IBD
		Primary	common	CVID
		Secondary	Solid cancer	S/P Wilms tumor
		Primary	common	CVID
		Primary	Immunodeficiency	Ataxia-Telangectasia
		Secondary	Solid Organ Transplant	LTX, KTX
		Primary	Immunodeficency	Ataxia-Telangectasia
36	Gupta	Secondary	Solid Organ Transplant	KTX

© 2023 Senefeld JW et al. *JAMA Network Open.*

No.	Study	Primary or Secondary	Category	Condition
		Secondary	Solid Organ Transplant	KTX
		Secondary	Solid Organ Transplant	KTX
		Secondary	Solid Organ Transplant	KTX
		Secondary	Solid Organ Transplant	KTX
		Secondary	Solid Organ Transplant	KTX
		Secondary	Solid Organ Transplant	KTX
		Secondary	Solid Organ Transplant	KTX
		Secondary	Solid Organ Transplant	KTX
		Secondary	Solid Organ Transplant	KTX
37	Halfmann PJ	Secondary	Infective	HIV
38	Hanssen JLJ	Secondary	Haematological Malignancy	CLL
39	Hartman (Prophylaxis)	Secondary	Haematological Malignancy	CLL
40	Hatzl	Secondary	Hematologic malignancy	NHL
		Secondary	Hematologic malignancy	NHL
41	Ho	Primary	humoral	CVID
		Primary	humoral	agammaglobulinemia
		Primary	humoral	agammaglobulinemia
		Primary	humoral	agammaglobulinemia
		Primary	humoral	agammaglobulinemia
42	Honjo	Secondary	Haematological Malignancy	CLL
43	Hovey	Primary	humoral	XLA
44	Hughes CM	Secondary	Haematological Malignancy	NHL
45	Iaboni	Primary	humoral	XLA
46	Jamir	Secondary	Solid Organ Transplant	LTX
47	Jassem J	Secondary	Haematological Malignancy	NHL
48	Jiang J	Secondary	Solid Organ Transplant	KTX
49	Jin H	Primary	humoral	XLA
		Primary	humoral	XLA
		Primary	humoral	XLA

© 2023 Senefeld JW et al. *JAMA Network Open.*

No.	Study	Primary or Secondary	Category	Condition
50	Karaolidou F	Secondary	Haematological Malignancy	ALL
51	Karatas	Secondary	Haematological Malignancy	NHL
52	Katz-Greenberg	Secondary	Solid Organ Transplant	KTX
		Secondary	Solid Organ Transplant	KTX
		Secondary	Solid Organ Transplant	KTX
		Secondary	Solid Organ Transplant	KTX
53	Keitel	Primary	common	SCID
54	Kemp	Secondary	Haematological Malignancy	NHL
55	Kenig	Secondary	Haematological Malignancy	NHL
		Secondary	Haematological Malignancy	NHL
		Secondary	Haematological Malignancy	NHL
		Secondary	Haematological Malignancy	NHL
		Secondary	Haematological Malignancy	CLL
56	Ketels	Secondary	Infective	HIV, HBV, Lue
57	Khan AM	Secondary	Haematological Malignancy	MM
58	Khatamzas	Secondary	Haematological Malignancy	NHL
59	Khatri	Secondary	Organ Transplant	HRT
60	Kluger	Secondary	Organ Transplant	KTX
		Secondary	Organ Transplant	KTX
		Secondary	Organ Transplant	KTX
61	Kremer AE	Secondary	Haematological Malignancy	ALL
		Secondary	Haematological Malignancy	NHL
		Secondary	Haematological Malignancy	NHL
62	Kutzler	Secondary	Organ Transplant	kidney
		Secondary	Organ Transplant	kidney
63	Lancman	Secondary	Haematological Malignancy	ALL
64	Lang-Meli J	Primary	common	CVID, BREAST CANCER, ACUTE CEREBELLITIS

© 2023 Senefeld JW et al. *JAMA Network Open.*

No.	Study	Primary or Secondary	Category	Condition
		Primary	common	CVID
		Primary	common	CVID
		Primary	common	CVID
		Primary	common	CVID
		Primary	common	CVID
		Primary	common	CVID
		Primary	humoral	XLA
		Primary	humoral	XLA
		Primary	humoral	XLA
		Primary	humoral	XLA
		Primary	humoral	Hyper IgM
		Primary	common	CVID
		Primary	common	CVID
		Primary	common	CVID
65	Lazzari L	Secondary	Haematological Malignancy	ALL
66	Lemus HN	Primary	common	CVID, BREAST CANCER
67	Lima	Secondary	Organ Transplant	HTX
		Secondary	Organ Transplant	HTX
68	Lindemann 2021a (J Med Virol)	Secondary	Organ Transplant	not specified
		Secondary	Organ Transplant	KTX
69	Ljungquist O	Secondary	Haematological Malignancy	not specified
		Secondary	Haematological Malignancy	not specified
		Secondary	Haematological Malignancy	not specified
		Secondary	Haematological Malignancy	not specified
		Secondary	Haematological Malignancy	not specified
		Secondary	Haematological Malignancy	not specified
		Secondary	Haematological Malignancy	not specified
		Secondary	Haematological Malignancy	not specified
		Secondary	Haematological Malignancy	not specified
		Secondary	Haematological Malignancy	not specified
		Secondary	Haematological Malignancy	not specified

© 2023 Senefeld JW et al. *JAMA Network Open.*

No.	Study	Primary or Secondary	Category	Condition
		Secondary	Haematological Malignancy	not specified
		Secondary	Haematological Malignancy	not specified
70	London	Primary	humoral	Hypogammaglobulinemia and B-cell alymphocytosis
71	Lubnow	Secondary	Haematological Malignancy	Hemophagocytic Lymphohistiocytosis
72	Luetkens	Secondary	Haematological Malignancy	MM
73	Madariaga	Secondary	Haematological Malignancy	HSCT
		Secondary	Solid Organ Transplant	LTX
74	Malsy	Secondary	Haematological Malignancy	NHL
75	Martens	Secondary	Solid Organ Transplant	HTX
76	Martínez-Barranco P	Secondary	Haematological Malignancy	NHL
		Secondary	Haematological Malignancy	NHL
		Secondary	Haematological Malignancy	NHL, IBD
		Secondary	Haematological Malignancy	NHL
77	Martinez-Chincilla	Secondary	Haematological Malignancy	NHL
		Secondary	Haematological Malignancy	NHL
78	Martinot	Secondary	Haematological Malignancy	CLL
79	McKemey	secondary	Autoimmune Disorder	rheumatoid arthritis
80	Mehta	Secondary	Solid Organ Transplant	KTX, HIV
		Secondary	Solid Organ Transplant	KTX, HIV
81	Mendes-Correa	Secondary	Haematological Malignancy	NHL
82	Meyts	Secondary	Kidney transplant	
		Primary	humoral	agammaglobulinemia
		Primary	humoral	CVID
		Primary	humoral	agammaglobulinemia
		Primary	humoral	CVID
		Primary	humoral	CVID
83	Milosevic	Primary	humoral	XLA
84	Mira	Primary	humoral	XLA

© 2023 Senefeld JW et al. *JAMA Network Open.*

No.	Study	Primary or Secondary	Category	Condition
85	Mohseni	Secondary	Solid Organ Transplant	LTX
86	Monrad	Secondary	Haematological Malignancy	CLL
87	Moore	Secondary	Haematological Malignancy	NHL
88	Moutinho-Pereira	Secondary	Autoimmune Disorder	SLE
89	Naeem	Secondary	Solid Organ Transplant	KTX
		Secondary	Solid Organ Transplant	KTX
		Secondary	Solid Organ Transplant	KTX
90	Nguyen	Secondary	Solid Organ Transplant	LTX
91	Niu	Secondary	Haematological Malignancy	ALL
92	Nussenblatt	Secondary	Haematological Malignancy	ALL
93	Nyström	Primary	humoral	XLA
94	Oliva	Secondary	Haematological Malignancy	NHL
		Secondary	Haematological Malignancy	NHL
		Secondary	Haematological Malignancy	NHL
		Secondary	Haematological Malignancy	NHL
		Secondary	Haematological Malignancy	NHL
		Secondary	Haematological Malignancy	NHL
95	Ordaya EE	Secondary	Haematological Malignancy	NHL
96	Ormazabal Velez	Secondary	Haematological Malignancy	NHL
		Secondary	Haematological Malignancy	IMA, BPCO, MCI STAGE IV
97	Pal	Secondary	Hematologic malignancy	LNH
		secondary	Hematologic malignancy	HSCT
		secondary	Hematologic malignancy	HSCT
		Secondary	Hematologic malignancy	ALL
		secondary	Hematologic malignancy	NHL
		Secondary	Hematologic malignancy	NHL

© 2023 Senefeld JW et al. *JAMA Network Open.*

No.	Study	Primary or Secondary	Category	Condition
98	Pommeret F	Secondary	Haematological Malignancy	ALL
		Secondary	Haematological Malignancy	NHL
		Secondary	Haematological Malignancy	FL
		Secondary	Haematological Malignancy	CLL
		Secondary	Haematological Malignancy	FL
99	Prasad	Secondary	Haematological Malignancy	NHL
100	Rahman	Secondary	Organ Transplant	Kidney
		Secondary	Organ Transplant	kidney
		Secondary	Organ Transplant	kidey
		Secondary	Organ Transplant	kidney
		Secondary	Organ Transplant	kidney
		Secondary	Organ Transplant	kidney
		Secondary	Organ Transplant	kidney
		Secondary	Organ Transplant	heart
		Secondary	Organ Transplant	liver
		Secondary	Organ Transplant	liver
		Secondary	Organ Transplant	liver
		Secondary	Organ Transplant	liver
		Secondary	Organ Transplant	kidney
101	Reuken	Secondary	Hematologic malignancy	NHL
102	Rem	Secondary	Autoimmune Disorder	MIXED COLLAGEN VASCULAR DISEASE
103	Ribeiro	Primary	common	CVID
104	Rnjak	Secondary	Haematological Malignancy	NHL
105	Rodriguez J	Secondary	Haematological Malignancy	MM
106	Rodriguez-Pla	Secondary	Autoimmune Disorder	Granulomatosis with polyangiitis
107	Rüfenacht	Secondary	Autoimmune Disorder	MIXED COLLAGEN VASCULAR DISEASE

© 2023 Senefeld JW et al. *JAMA Network Open.*

No.	Study	Primary or Secondary	Category	Condition
108	Schenker C	Secondary	Haematological Malignancy	CLL
109	Schreiber	Secondary	Solid Organ Transplant	HTX
110	Sepulcri	Secondary	Haematological Malignancy	NHL
111	Shankar R	Secondary	Haematological Malignancy	ALL
112	Spinicci M	Secondary	Haematological Malignancy	HIV, NHL
113	Steiner	Secondary	Autoimmune Disorder	GLILD, thalassemia minor
		Secondary	Solid cancer	none (thymectomy due to thymoma in 2016)
114	Szwebel	Secondary	Haematological Malignancy	HIV, NHL
115	Taha	Secondary	Haematological Malignancy	NHL
116	Trimarchi	Secondary	Solid Organ Transplant	KTX
117	Truong	Secondary	Hematologic malignancy	ALL
118	Van Damme	Primary	common	CVID
119	van Oers	Primary	common	SCID
120	Wang	Secondary	Hematologic malignancy	MM
121	Wright	Secondary	Haematological Malignancy	NHL
122	Yee	Secondary	Autoimmune Disorder	Sjogren syndrome
123	Zhang LB	Secondary	Autoimmune Disorder	SLE
124	Zhang LL	Secondary	Haematological Malignancy	CML
125	Zimmerli	Secondary	Haematological Malignancy	CLL
126	Zimmermann	Secondary	Autoimmune Disorder	idiopathic pulmonary fibrosis

© 2023 Senefeld JW et al. *JAMA Network Open.*

eTable 7. Individual Patient Data (Part VIII)					
No.	Study	anti-CD20	Anti-Spike mAbs (YES/No)	Pre/ post/ concomitatnt	type of anti-Spike mAb
1	Abid		No		
			No		
2	Adedoyin O		No		
			No		
3	Antony		No		
4	Avanzato		No		
5	Baang	Y	No		
6	Bakhsh A		No		
7	Balashov		No		
8	Basheer M	Y	No		
9	Bayrak		No		
10	Belcari	Y	No		
11	Bosnjak B		No		
12	Bronstein	Y	Yes	pre	BAM
13	Bruiners N	Y	No		
14	Buckland				
15	Casarola G		no		
16	Chen L		No		
17	Choudhury A		No		
18	Christensen J		No		
19	Cinar O		No		
20	Clark		No		
21	Colombo	Y	No		
22	Cusi MG		No		
			No		
			No		
23	D'abramo	Y	No		
		Y	Yes	POST	CAS+IMD
		Y	No		
		Y	No		
		Y	No		
		Y	Yes	PRE	CAS+IMD
		Y	Yes	pre	BAM+ETE
24	Dale M		Yes	pre	BAM
			Yes	pre	BAM
25	Delgado-Fernandez		No		
		Y	No		
		Y	No		
26	Dell'Isola		No		
27	Deveci B		No		

© 2023 Senefeld JW et al. *JAMA Network Open.*

No.	Study	anti-CD20	Anti-Spike mAbs (YES/No)	Pre/ post/ concomitatnt	type of anti-Spike mAb
			No		
			No		
28	Di Palma		No		
29	Erber J		No		
30	Ferrari		No		
			No		
			No		
			No		
			No		
			No		
			No		
31	Franchini	Y	No		
			No		
			No		
			No		
			Yes	pre	CAS+IMD
			No		
		Y	No		
		Y	No		
			No		
32	Fung		No		
			No		
			No		
			No		
33	Furlan		No		
			No		
			No		
			No		
34	Gattuso G		No		
35	Gordon O		No		
			No		
			No		
			No		
			No		
			No		
			No		
			No		
			No		
36	Gupta		No		
			No		
			No		
			No		

© 2023 Senefeld JW et al. *JAMA Network Open.*

No.	Study	anti-CD20	Anti-Spike mAbs (YES/No)	Pre/ post/ concomitatnt	type of anti-Spike mAb
			No		
			No		
			No		
			No		
			No		
			No		
37	Halfmann PJ		Yes	pre\post	BAM
38	Hanssen JLJ		No		
39	Hartman (Prophylaxis)		No		
40	Hatzl				
41	Ho				
42	Honjo		No		
43	Hovey		No		
44	Hughes CM		No		
45	Iaboni		No		
46	Jamir		No		
47	Jassem J	Y	No		
48	Jiang J		No		
49	Jin H		No		
			No		
			No		
50	Karaolidou F		No		
51	Karatas		No		
52	Katz-Greenberg		No		
			No		
			No		
			No		
53	Keitel		No		
54	Kemp		No		
55	Kenig		No		
			No		
			No		
			No		
			No		
56	Ketels		No		
57	Khan AM		No		

© 2023 Senefeld JW et al. *JAMA Network Open.*

No.	Study	anti-CD20	Anti-Spike mAbs (YES/No)	Pre/ post/ concomitatnt	type of anti-Spike mAb
58	Khatamzas		No		
59	Khatri		No		
60	Kluger		No		
			No		
			No		
61	Kremer AE		No		
			No		
			No		
62	Kutzler				
63	Lancman	Y	No		
64	Lang-Meli J		No		
			No		
			No		
			No		
			No		
			No		
			No		
			No		
			No		
			No		
			No		
			No		
			No		
			No		
			No		
65	Lazzari L		No		
66	Lemus HN		No		
67	Lima		No		
			No		
68	Lindemann 2021a (J Med Virol)	Y	No		
		Y	No		
69	Ljungquist O		No		

© 2023 Senefeld JW et al. *JAMA Network Open.*

No.	Study	anti-CD20	Anti-Spike mAbs (YES/No)	Pre/ post/ concomitatnt	type of anti-Spike mAb
70	London		No		
71	Lubnow		No		
72	Luetkens		No		
73	Madariaga		No		
			No		
74	Malsy	Y	No		
75	Martens		No		
76	Martínez-Barranco P		No		
			No		
			No		
		Y	No		
77	Martinez-Chincilla	Y	No		
		Y	No		
78	Martinot		No		
79	McKemey	Y	No		
80	Mehta		No		
			No		
81	Mendes-Correa		No		
82	Meyts				
83	Milosevic		No		
84	Mira		No		
85	Mohseni		Yes	post	non specificati
86	Monrad		No		
87	Moore	Y	No		
88	Moutinho-Pereira		No		
89	Naeem		No		
			No		
			No		
90	Nguyen		No		
91	Niu		No		
92	Nussenblatt		No		
93	Nyström		No		

© 2023 Senefeld JW et al. *JAMA Network Open.*

No.	Study	anti-CD20	Anti-Spike mAbs (YES/No)	Pre/ post/ concomitatnt	type of anti-Spike mAb
94	Oliva	Y	no		
			No		
			No		
			No		
			No		
			No		
95	Ordaya EE		No		
96	Ormazabal Velez		No		
		Y	No		
97	Pal		No		
			No		
			No		
			No		
			No		
			No		
98	Pommeret F		No		
			No		
			No		
			No		
			No		
99	Prasad	Y	No		
100	Rahman				
101	Reuken				
102	Rem	Y	No		
103	Ribeiro		No		
104	Rnjak		No		
105	Rodriguez J		No		
106	Rodriguez-Pla		No		
107	Rüfenacht	Y	No		

© 2023 Senefeld JW et al. *JAMA Network Open.*

No.	Study	anti-CD20	Anti-Spike mAbs (YES/No)	Pre/ post/ concomitatnt	type of anti-Spike mAb
108	Schenker C		No		
109	Schreiber		No		
110	Sepulcri	Y	No		
111	Shankar R		No		
112	Spinicci M		No		
113	Steiner		No		
			No		
114	Szwebel		No		
115	Taha	Y	Yes	post	BAM+ETE
116	Trimarchi		No		
117	Truong				
118	Van Damme		No		
119	van Oers		No		
120	Wang				
121	Wright		No		
122	Yee		No		
123	Zhang LB		No		
124	Zhang LL		No		
125	Zimmerli		No		
126	Zimmermann		Yes	concomitant	BAM+ETE

© 2023 Senefeld JW et al. *JAMA Network Open.*
