# Supplementary material for: COVID-19 Convalescent Plasma for the Treatment of Immunocompromised Patients: A Systematic Review and Meta-analysis
Source: JAMA Netw Open. 2023 Jan 12;6(1):e2250647. doi: 10.1001/jamanetworkopen.2022.50647 (PMC9857047; doi:10.1001/jamanetworkopen.2022.50647)
Supplement: Supplement 2. — Data-Sharing Statement [file jamanetwopen-e2250647-s002.pdf]

# Data Sharing Statement

Senefeld. COVID-19 Convalescent Plasma for the Treatment of Immunocompromised Patients: A Systematic Review and Meta-analysis. *JAMA Netw Open*. Published January 12, 2023. doi:10.1001/jamanetworkopen.2022.50647

**Data**
**Data available:** No

**Additional Information**
**Explanation for why data not available:** This secondary research did not generate original data, which remain available at the cited references.
